# Dynamic changes in species richness and community diversity of symbiotic bacteria in five reproductive morphs of cotton aphid *Aphis gossypii* Glover (Hemiptera: Aphididae)

**DOI:** 10.3389/fmicb.2022.1086728

**Published:** 2023-01-12

**Authors:** Ruifang Chen, Junyu Luo, Xiangzhen Zhu, Li Wang, Kaixin Zhang, Dongyang Li, Xueke Gao, Lin Niu, Ningbo Huangfu, Xiaoyan Ma, Jichao Ji, Jinjie Cui

**Affiliations:** ^1^Zhengzhou Research Base, State Key Laboratory of Cotton Biology, School of Agricultural Sciences, Zhengzhou University, Zhengzhou, China; ^2^State Key Laboratory of Cotton Biology, Institute of Cotton Research, Chinese Academy of Agricultural Sciences, Anyang, China; ^3^Western Agricultural Research Center, Chinese Academy of Agricultural Sciences, Changji, China

**Keywords:** symbiotic bacteria, 16S rRNA, *Arsenophonus*, *Aphis gossypii*, reproductive polymorphism

## Abstract

**Introduction:**

Reproductive polymorphism and symbiotic bacteria are commonly observed in aphids, but their interaction remains largely unclear. In polymorphic aphid species (*Aphis gossypii*), offspring of parthenogenetic females (PFs) develops into sexuparae which produces gynoparae and males successively. Gynoparae further produces sexual females (SFs), and these sexual females mate with males to produce offspring.

**Methods:**

In this study, we investigated the dynamic changes of symbiotic bacteria during the above-mentioned five reproductive morph switch in *A. gossypii* via 16S rRNA sequencing technology.

**Results:**

The results showed that species richness and community diversity of symbiotic bacteria in males were the highest. Proteobacteria was absolutely dominant bacterial phylum (with relative abundance of more than 90%) in the five reproductive morphs of *A. gossypii*, and *Buchnera* was absolutely dominant genus (with relative abundance of >90%), followed by *Rhodococcus, Pseudomonas,* and *Pantoea*. Male-killing symbiont *Arsenophonus* presented the highest relative abundance in gynoparae, a specific morph whose offsprings were exclusively sexual females. Both principal component analysis (PCA) and clustering analysis showed trans-generation similarity in microbial community structure between sexuparae and sexual females, between PFs and gynoparae. PICRUSt 2 analysis showed that symbiotic bacteria in the five reproductive morphs were mainly enriched in metabolic pathways.

**Discussion:**

Reproductive morph switch induced by environmental changes might be associated with bacterial community variation and sexual polymorphism of aphids. This study provides a new perspective for further deciphering the interactions between microbes and reproductive polymorphism in host aphids.

## 1. Introduction

Insects are by far the most diverse and abundant populations of animals (Simon et al., 2010). The exoskeletons, intestines, hemolymph, and cells of insects are permanently colonized by a variety of microorganisms (Zhang et al., 2015). Endosymbiotic bacteria profoundly influence insect survival and drive the coevolution between plants and herbivores (Schirmer et al., 2008; Kikuchi et al., 2012) by participating in nutrient metabolism, growth, and reproduction, environmental adaptation, and drug resistance (Douglas, 1998; Dillon and Dillon, 2004; Douglas, 2009; Adams et al., 2010; Michalkova et al., 2014). For example, co-occurring *Wolbachia* and *Spiroplasma* endosymbionts can modulate immune signaling of *Drosophila melanogaster* against infection by virulent pathogen bacterium *Photorhabdus luminescens* (Shokal et al., 2016). Sublethal concentration of sulfoxaflor has intergenerational excitatory effect on cotton aphids G1 and G2 and can significantly alter their bacterial community and abundance (Shang et al., 2021).

Cotton, one of the most economically important crops in the world, is infested with various pests during the production such as bollworms, thrips, and aphids. For decades, the Bt cotton (genetically engineered plants expressing pesticidal Cry toxins derived from *Bacillus thuringiensis*) has been widely adopted to effectively suppress caterpillars. However, the population number of non Bt-targeted pests, like mirid bug and cotton aphid, are increasing due to the reduced levels of induced defensive terpenoids and drops in insecticide use (Lu et al., 2010; Hagenbucher et al., 2013). The cotton aphid *Aphis gossypii* is a worldwide agricultural pest distributed in 171 countries and feeds by sucking sap from plants, causing leaf curling and deformation, which greatly hinders effective photosynthesis of plants (Henneberry et al., 2000; Gupta, 2001; Schirmer et al., 2008; Ji et al., 2021). *A*. *gossypii* has a wide host range covering nearly 900 plant species belonging to 116 families such as Malvaceae, Asteraceae, Cucurbitaceae, Rutaceae, and Rosaceae (Ma et al., 2019). In the past 40 years, *A*. *gossypii* has progressively spread widely in the main cotton-producing regions of China, leading to a rapid increase in infected areas and heavy economic losses (Dedryver et al., 2010). Hence, it is urgent to develop new strategies for controlling this piercing-sucking pest.

Symbiotic bacteria exist in almost all the aphid species, and considerable studies have revealed their vital contributions for host aphids including nutrition supply, anti-parasitism, drug resistance, and body color plasticity (Douglas, 1998; Tsuchida et al., 2010; Donald et al., 2016; Li et al., 2018). In pea aphid *Acyrthosiphon pisum*, symbiotic bacterium *Serratia symbiotica* can promote its host growth and development by enhancing host fatty acid metabolism (Zhou et al., 2021). Infection with symbionts such as *Rickettsia* confers pea aphid the resistance to the attack from parasitic wasp *Aphidius ervi* by causing high mortality of developing parasitoid larvae (Oliver et al., 2003). *Sitobion miscanthi* infected with *Hamiltonella defensa* is more resistant to imidacloprid, acetamiprid, and cyantraniliprole than those uninfected (Li et al., 2021). Facultative endosymbiont *Rickettsiella* can alter aphid body color from red to green in natural populations by interfering with host pigment biosynthesis to regulate blue-green pigment production (Tsuchida et al., 2010). Facultative symbionts may also be limited by their location, persistence of host lineage, and horizontal transfer of symbionts to affect aphid host adaptation (Russell et al., 2003; Moran and Dunbar, 2006; Hansen and Moran, 2014). Studies have shown that the co-infection of *Hamiltonella defensa* and *Arsenophonus* sp. helps enhance its performance to promote the adaptability of cotton aphids (Ayoubi et al., 2020). Symbiotic bacteria vary on different hosts, but host specificity of *A*. *gossypii* is not controlled by specific bacterial symbionts or *Buchnera* abundance (Gauthier et al., 2015; Guo et al., 2022). Host diet and immune aging also affect symbiont populations (Laughton et al., 2014; Zhang et al., 2019).

Reproductive polymorphism, the capability of reproducing sexually and asexually upon environment changes, is an adaptation strategy commonly observed in multiple pests, especially aphids, and it can improve pest survival largely during the evolution (Liu et al., 2014; Ji et al., 2021). For example, in the termite *Reticulitermes speratus*, neotenic queens are produced by monogamous reproduction by the queen, while other colony members are produced by sexual reproduction by the queen (Yashiro and Matsuura, 2014). Locusts are one of the most complex examples of phenotypic plasticity, with individuals becoming swarming locusts due to changes in density, and two modes of reproduction exist in locusts, namely sexual and asexual reproduction, to adapt to environmental changes (Hamilton, 1953; Little et al., 2017; Foquet et al., 2021). Asexual reproduction of *A*. *gossypii* is conducted in spring and summer, in which parthenogenetic females (PFs) produces plentiful nymphs *via* parthenogenesis. In late autumn, gynoparae flies to the winter host and produces the sex females which mates with the subsequently migratory males to lay fertilized eggs (Simon et al., 2010; Ji et al., 2021). However, the interactions between reproductive plasticity and symbiotic bacteria in insects remain largely unknown. Stouthamer et al. (1990) found that the treatment of asexual *Trichogramma* wasps with antibiotics would lead to their recovery of sexual reproduction. It is precisely because PI *Wolbachia* symbiosis caused their parthenogenesis (Stouthamer et al., 1990; Russell and Stouthamer, 2011). The presence or absence of environmental stressors and bacteria may affect phenotypes (reproductive strategies and morphological characteristics; Reyes et al., 2019; Carpenter et al., 2021). When *Acyrthosiphon pisum* was exposed to the aphid alarm pheromone (E) -β-farnesene (EBF), aphids with defense against *Hamilton* or *Serratia* symbiotic infection increased reproduction (Barribeau et al., 2010). Some secondary symbionts in *Acyrthosiphon pisum* are frequently lost during sexual reproduction of their hosts and regained during parthenogenesis (Peccoud et al., 2014). It is worthwhile to explore symbiotic bacteria dynamics during the reproductive morph switch in cotton aphid due to its most valuable available resources including chromosome-level genome (Zhang et al., 2022), standardized sexual reproduction induction methods (the method of inducing sexual reproduction in cotton aphids by changing photoperiod and temperature has been well established, and the morphological characteristics of different reproductive types of cotton aphids have been clarified; Kwon and Kim, 2017; Ji et al., 2021; Huangfu et al., 2022), high-efficiency RNAi technology (Pan et al., 2018; Ullah et al., 2020; Zeng et al., 2021; Li et al., 2022; Peng et al., 2022).

To explore the interaction between reproductive polymorphisms and symbiotic bacteria within aphids, this study investigated and compared the bacterial community composition and relative abundance reproductive morphs of *A*. *gossypii* using 16S rDNA gene sequencing technology. The association between reproductive polymorphisms and aphid symbiotic bacteria was further understood by functional prediction of the bacteria as well as cluster analysis. This study provides new perspectives on the effect of symbiotic bacteria on aphid reproduction, and can open the way to the formulation of novel aphid control strategies.

## 2. Materials and methods

### 2.1. Insect rearing

The *A*. *gossypii* used in this study was laboratory-purified multigenerational populations, which were reared on cotton variety CCRI49 with population densities and plant growth routinely monitored. All aphids were produced on CCRI49. A total of 32 cotton plants were used, with 20 aphids on each plant. PFs were reared at 25°C ± 1°C with 75% ± 5% relative humidity and a photoperiod of 16-h light: 8-h darkness. Offsprings of PFs (initial generation) were developed into sexuparae (generation 1), which gave birth to gynoparae and males (generation 2) successively under controlled short-day conditions of 18°C ± 1°C, 75 ± 5% relative humidity, and 8-h lightness: 16-h darkness photoperiod, in which gynoparae exclusively produced sexual females (generation 3; Kwon and Kim, 2017; Ji et al., 2021).

### 2.2. Sampling, DNA extraction, and 16S rRNA amplification sequencing

Five reproductive morphs including PFs, sexuparae, gynoparae, sexual females (SFs), and males obtained according to protocols above were soaked in 75% ethanol solution for 5 min and then washed 3 times with ultrapure water, respectively. The experiments were conducted with 4 biological replicates and at least 100 aphids per replicate. Total DNA was extracted using the Blood Genomic DNA Extraction Kit (Tiangen Biochemical, Beijing). DNA concentration of aphids was detected with a Nanodrop 2000C (Thermo Fisher Scientific, United States) micro spectrophotometer, and band integrity was determined through 1.2% gel electrophoresis. The V3-V4 variable region sequence of 16S rDNA gene was amplified using the GeneAmp9700 thermal cycling PCR system (Applied Biosystems, United States) with universal primer pair (338F:5’-ACTCCTACGGGAGGCAGCAG-3′, 806R:5’-GGACTACHVGGGTWTCTAAT-3′; Gao et al., 2021). Miseq library construction method: (1) Addition of official Illumina junction sequences to the outer end of the target region by PCR. (2) Recovery of PCR products by cutting gels using a gel recovery kit. (3) Elution with Tris–HCl buffer and detection by 2% agarose electrophoresis. (4) Denaturation by sodium hydroxide to produce single-stranded DNA fragments. Reagent: TruSeqTM DNA Sample Prep Kit. The PCR was conducted with 10 ng DNA used as template in reaction system containing 0.8 μL (5 μmol/l) each primer (338F/806R), 2 μL (2.5 mmol/l) dNTPs, 4 μL (5× FastPfu) buffer, 0.4 μL FastPfu polymerase, and ddH_2_O was added to reach 20 μL reaction system volume. Amplification procedure: 95°C pre-denaturation for 3 min, 35 cycles (95°C denaturation for 30 s, 55°C annealing for 30 s, 72°C extension for 30 s), and finally 72°C extension for 10 min.

### 2.3. High-throughput sequencing and statistical analysis

Sequencing of symbiotic bacteria was performed *via* Illumina MiSeq PE 300 platform (Meiji Biomedical Technology, Shanghai). The raw FASTQ data were subjected to quality control by previously reported method (Zhao et al., 2019). Based on OTUs (operational taxonomic units), the sequences of symbiotic bacteria were analyzed using UPARSE software (version 7.1), and the clustering analysis was performed according to the sequence similarity of 97%. Symbiotic bacteria were classified using RDP classifier^1^ by aligning against the Silva (SSU128) 16S rRNA database with an alignment threshold of 70%. The Wilcoxon rank-sum test was performed to determine difference significance, followed by the probability (*p*-value) correcting *via* FDR (false discovery rate) approach. Significant differences in bacterial relative abundance between sample groups were investigated at the OTU level with the thresholds of FDR < 0.01 and *p* < 0.05. The raw sequence data were deposited in the GenBank SRA database with accession number of PRJNA877298. Unweighted pair group method arithmetic mean (UPGMA) was used to analyze the microbial community structure of five reproductive morphs of *A*. *gossypii*. The functions of symbiotic bacteria in cotton aphids were predicted using software PICRUSt 2, and the abundance of each functional category was calculated according to the KEGG database and the OTU abundance information table (Xue et al., 2021a). Statistical analysis was performed by one-way ANOVA using SPSS software to investigate significant differences in symbiotic bacterial abundance and community diversity among the five reproductive forms. Columns (Mean ± SE) with the same letters are not significantly different (Duncan test; *p* > 0.05). Gradient diluted plasmid concentrations were converted into the copy number of the bacteria and made into the standard curve of the bacteria. The CT value of the sample was substituted into the curve equation to calculate the sample copy number. The copy numbers of different samples were normalized to make them comparable. Due to the large number of copies, we performed Log conversion.

### 2.4. Phylogenetic analysis of *Buchnera* and *Arsenophonus*

The homologous nucleic acid sequences of two important symbiotic bacteria *Buchnea* and *Arsenophonus* in aphids were searched against the NCBI (National Center for Biotechnology Information) nucleotide (nr) database *via* blastn strategy (Chen et al., 2015). Fifteen 16S rRNA sequences of *Buchnera* and *Arsenophonus* obtained from GenBank were used to construct phylogenetic trees through MEGA7.0 software by neighbor-joining method (1,000 bootstraps), respectively (Kumar et al., 2016).

### 2.5. Quantification of symbiotic bacteria

The quantitative polymerase chain reaction (qPCR) was used to investigate the copy number of symbiotic bacteria in five reproductive aphid morphs (Xue et al., 2021b). DNA extracted from five reproductive morphs of *A*. *gossypii* was diluted 10 folds and used as template for PCR amplification. The specific primer pairs (*Buchnera*, F: 5’-AGTATCGTAGAGGGAGGTA-3′, R: 5’-CTTTCGCCACAGGTATTC-3′; *Arsenophonus*, F: 5’-CATTCAAGACTGGTTAGC-3′, R: 5’-TATTCCTCCACATCTCTAC-3′) were used for qPCR. The target gene was transferred into a plasmid using the cloning vector pEASY-T3 (Transgenic Biotechnology, China), and preparation of bacterial standard curve by diluting 6 gradients of plasmid concentration. The target sequence in the standard vector was confirmed by sequencing. Each reaction plate has a corresponding standard curve. All qPCR reactions were repeated three times on each DNA sample to ensure that the specificity of each primer was verified. The qPCR was performed on Step OnePlus™ Real-Time PCR System (Applied Biosystems, United States). The 10 μL reaction system contained 1 μL of template DNA, 5 μL 2 × TransStart Green qPCR SuperMix (TransGen Biotech, China), 0.2 μL of each forward primer and reverse primer, and 3.6 μL of ddH_2_O. The qPCR reaction conditions were as follows: 94°C for 30 s, followed by the 40 cycles of 94°C for 5 s and 60°C for 30 s. The specificity of amplification was verified by melting curve.

## 3. Results and discussion

### 3.1. Overview of *Aphis gossypii* microbiota

**Figure 1:**
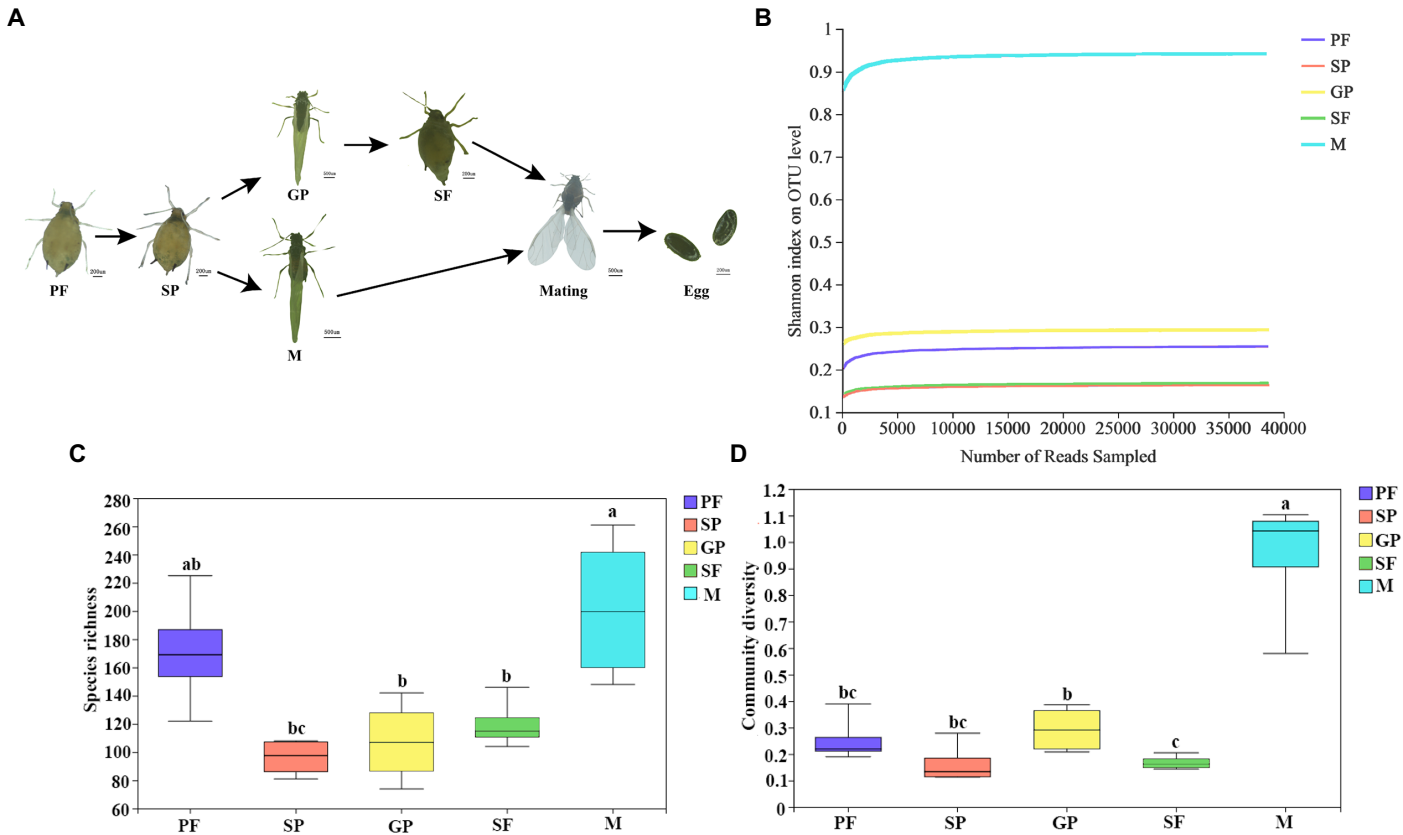
Schematic of life cycle of five morphs and dynamics of symbiotic bacterial species richness and community diversity in *A*. *gossypii*. **(A)** Overview of life cycle of 5 aphid morphs. **(B)** Rarefaction curves of bacterial community Shannon index with four biological replicates for each morph at the OTU level. **(C)** Boxplot of species richness evaluated by OTUs number. **(D)** Bacterium community diversity evaluated by Shannon index. Different lower-case letters represent significant differences (*p* < 0.05) revealed by one-way ANOVA. PF, parthenogenetic females; SP, sexuparae; GP, gynoparae; SF, sexual females; and M, males.

Cotton aphid can switch between parthenogenesis and gamogenesis for reproduction upon environmental changes. Under short photoperiod and low temperature conditions, offsprings of PFs can develop into sexuparae (SP) which in turn produces gynoparae (GP) and males successively. Gynoparae merely produces sexual females (SFs), and these sexual females mate with males to lay fertilized eggs (Figure 1A). Microbial communities in these five reproductive morphs of cotton aphid were identified through 16S rDNA sequencing. A total of 1, 605,749 clean reads were obtained, and 1,898 OTUs were assigned with an average length of 428 bp. Rarefaction curves were used to examine the quality of the sequencing. The results showed that all the curves tended to saturate after the number of sequences reached 35, 000, indicating that most of the microbial species were captured in all the samples (Figure 1B). The average sequencing coverage rate of each sample was more than 99%, indicating that the experimental data accurately reflected the composition of most bacterial community in all samples. The Chao1 index showed significant differences between mother and offspring (PF vs. SP; SP vs. GP or males; and GP vs. SP), and similarly, significant differences were also found in other α diversity indices (ACE, Shannon, and Simpson; Supplementary Table S1).

Microbial community diversity was evaluated by α diversity, and significant differences in bacterial species diversity and richness were observed across different reproductive morphs of *A*. *gossypii*. Males exhibited the largest number of bacterial species with an average of 202 OTUs per sample, followed by PFs with an average of 171 OTUs per sample (Supplementary Table S1). The species richness of symbiotic bacteria in sexuparae, sexual females, and gynoparae was relatively low, with sexuparae exhibiting the lowest number of bacterial species with an average of 96 OTUs (Supplementary Table S1). It has been reported that aphids females have two X chromosomes whereas males have only one, while keeping the same diploid status of the remaining autosomes (Yan et al., 2020). Symbiotic bacteria species richness of *A*. *gossypii* declined in the switch from asexual to sexual reproduction (i.e.PF–SP–GP–SF–M; Figure 1C). With the reproductive morph transformation (switching from asexually PFs to sexual females and male through intermediate morphs SPe and GPe) of cotton aphid, community diversity of symbiotic bacteria fluctuated, and reached peak in males (Figure 1D). Our data showed that compared to the other four reproductive morphs, males exhibited higher bacterial species richness and community diversity (Figures 1C, D), which might be related to sex differences. Similar to our results, microbial diversity analysis of four species of eastern North American blackflies, *Simulium innoxium*, *Simulium dixiense*, *Simulium krebsorum*, and *Simulium slossonae*, showed that the bacterial diversity of males was higher than that of females (Tang et al., 2012).

### 3.2. Bacteria dynamics among five reproductive morphs of *Aphis gossypii*

**Figure 2:**
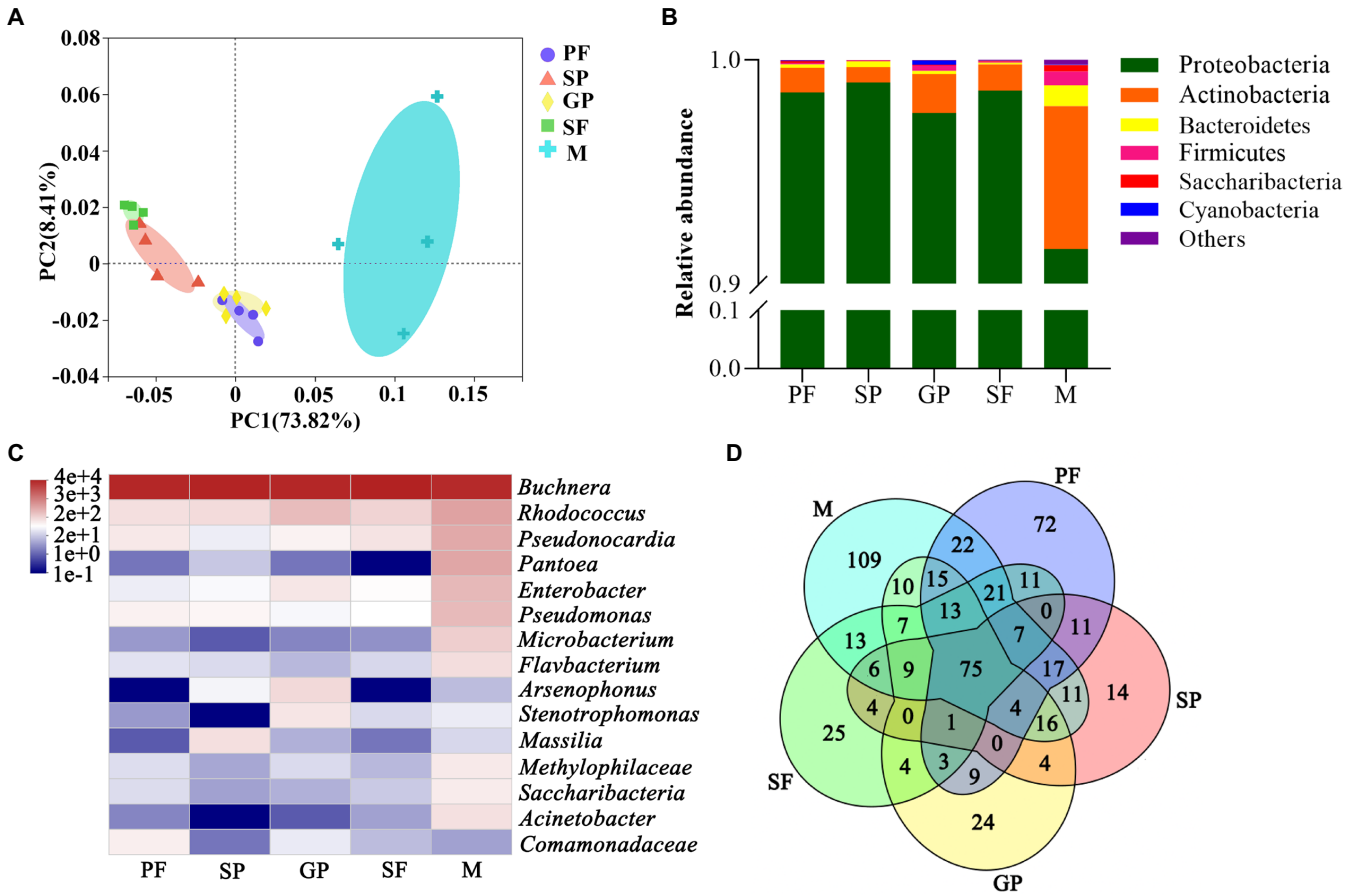
Dynamic changes of symbiotic bacterium communities during reproductive morph switch of *A*. *gossypii*. **(A)** Principal component analysis (PCA) of 20 samples from five reproductive morphs of *A*. *gossypii*. **(B)** Relative abundance of symbiotic bacteria in five reproductive morphs of *A*. *gossypii* at phylum level. **(C)** Heatmap of top 15 dominant microbial genera based on log2-transformed relative abundance. **(D)** Venn diagram of shared and exclusive bacteria in five reproductive morphs at species level. PF, parthenogenetic females; SP, sexuparae; GP, gynoparae; SF, sexual females; M, males.

Principal component analysis (PCA) showed that the scatter points corresponding to the five reproductive morphs of *A*. *gossypii* showed clustering with each other within groups, indicating a relatively good repeatability within groups and very similar sample data, while there was a good differentiation between groups. In addition, the distance between PFs and gynoparae, and between sexuparae and sexual females was closer, indicating that symbiotic bacterial composition of these four reproductive morphs of *A*. *gossypii* was similar mutually (Figure 2A).

At the phylum level, the relative abundance of bacteria differed among the five reproductive morphs of *A*. *gossypii* (Figure 2B). Proteobacteria was dominant in all samples with the highest relative abundance of 99.00% in sexuparae, followed by 98.63% in sexual females, 98.56% in PFs, 97.65% in gynoparae, and 91.56% (the lowest) in males (Supplementary Table S2). Compared with other four reproductive morphs, males displayed higher relative abundance of Actinobacteria (6.38%), Bacteroidetes (0.92%), Firmicutes (0.62%), Saccharibacteria (0.29%), and Cyanobacteria (0.025%; Supplementary Table S2). Our data showed that Proteobacteria was the most dominant phylum at all the cotton aphid taxonomic levels, which was consistent with several previous reports on other aphid species such as *Myzus persicae*, and *Sitobion avenae* (Alkhedir et al., 2015; Xu et al., 2021). The relative abundance of these bacterial phyla except Proteobacteria was all significantly higher in males than in other four reproductive morphs of *A*. *gossypii*.

**Figure 3:**
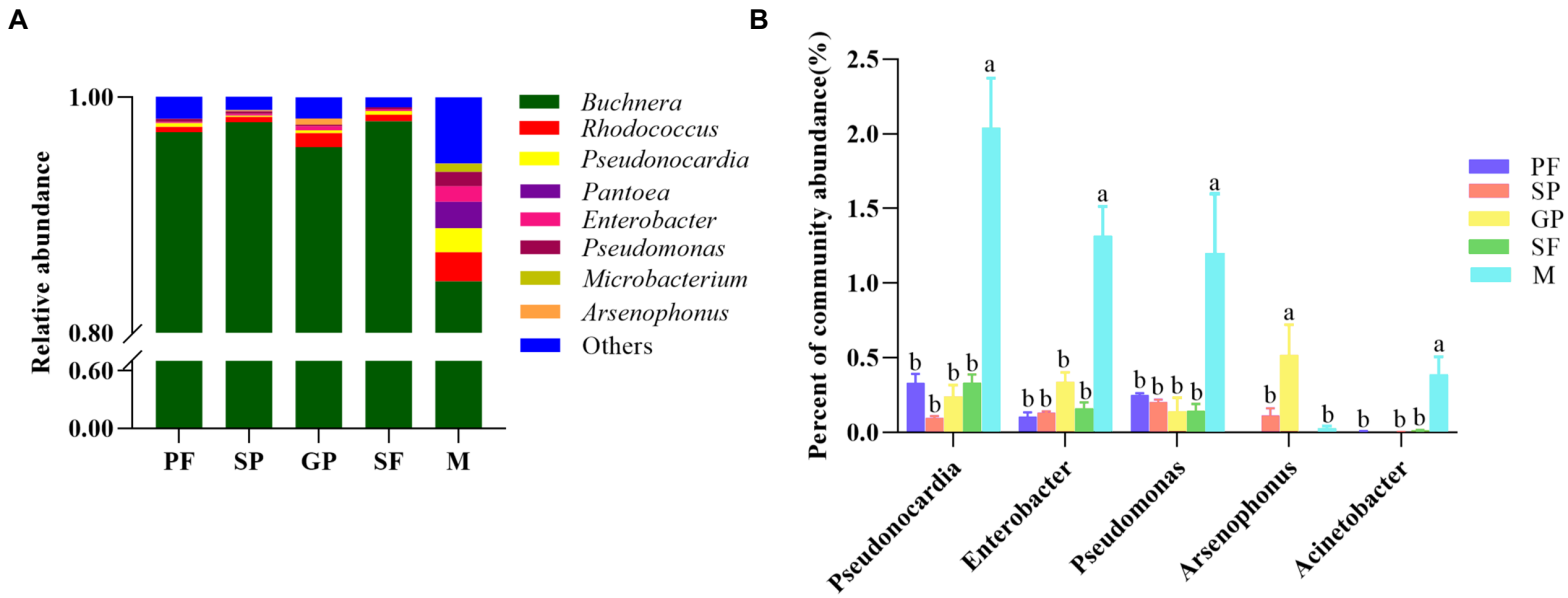
Relative abundance of microbial genera and its comparison across five reproductive morphs. **(A)** Relative abundance of major microbial genera in five reproductive morphs. **(B)** Comparison of relative abundance of five common secondary symbiotic bacteria in five reproductive morphs. Different lower-case letters represented significant differences (*p* < 0.05) based on one-way ANOVA test. PF, parthenogenetic females; SP, sexuparae; GP, gynoparae; SF, sexual females; M, males.

The dynamic abundance of the top 15 bacterial genera was visualized across the reproductive morphs of cotton aphid by heat maps (Figure 2C). Obviously, *Buchnera* was the dominant bacterial genus in all the five reproductive morphs with an average relative abundance of 94.60%. *Rhodococcus* (2.48%), *Pantoea* (2.22%), *Pseudonocardia* (2.04%), *Enterobacter* (1.32%), *Pseudomonas* (1.21%), *Microbacterium* (0.68%), *Flavobacterium* (0.44%), *Acinetobacter* (0.39%), *Methylophilaceae* (0.31%), and *Saccharibacteria* (0.29%) were relatively abundant in males, but their relative abundance was lower in other four morphs. Moreover, the relative abundance of *Arsenophonus* and *Stenotrophomonas* was the highest in gynoparae, whereas that of *Massilia* was the highest in sexuparae, and that of *Comamonadaceae* was the highest in PFs (Supplementary Table S3). Our results showed that the primary symbiotic bacterium *Buchnera* was absolutely dominant in all the five different reproductive morphs of *A*. *gossypii*, which was consistent with one previous report (Zhao et al., 2016). All aphids carry the obligate symbiotic *Buchnera*, and this bacterium can synthesize essential nutrients that aphids can not obtain from their diets (Douglas, 2003), playing an important symbiotic role in the growth, development and survival of *A*. *gossypii*. In this study, *Rhodococcus*, *Pseudonocardia*, *Pantoea*, and *Enterobacter* were also detected in all five reproductive morphs (Figure 3A), of which *Rhodococcus* is the second dominant bacterial genus among the five reproductive morphs of aphids. *Rhodococcus* can be isolated from the Madagascar hissing cockroach *Gromphadorhina portentosa* (Guzman and Vilcinskas, 2021). *Rhodococcus* has higher alcohol dehydrogenases, oxidases, P450 monooxygenases and other enzyme activities, mainly responsible for many organic matter biotransformation and biodegradation (Bell et al., 1998; Busch et al., 2019). Cytochrome P450 monooxygenases play an important role in the metabolism of *A*. *gossypii* and the detoxification of toxic plant substances (Gao et al., 2022). However, whether the P450 monooxygenase in *Rhodococcus* directly affects the metabolism of aphids is not clear (Franks et al., 2021) have found that diet treatment with *Rhodococcus* can raise diet conversion efficiency, thus increasing body size in black soldier fly larvae. Based on these findings, we hypothesized that *Rhodococcus* in cotton aphid might be also responsible for biotransformation and biosynthesis. In this study, the relative abundance of *Rhodococcus* in the winged gynoparae and males was significantly higher than that in the other three reproductive wingless aphids (Figures 2C, 3A). We speculate that the winged gynoparae and males need more energy to support flight than the wingless aphids. But the specific mechanism is not clear.

*Pseudonocardia* can produce a variety of secondary metabolites with antifungal and antibacterial bioactivities in ants to protect host from disease (Goldstein and Klassen, 2020). Based on this, we speculated that *Pseudonocardia* with high relative abundance in five reproductive morphs (Top 3 bacterium genus, Figures 2C, 3A) might prevent cotton aphids from the disease during the reproductive morph switch. *Pantoea* is widely distributed in water bodies, plants, soil, and insects, and this bacterium can also be transmitted vertically by the contamination of the egg surface in the stinkbug *Plautia stali* (Oishi et al., 2019). In host insects, *Pantoea* plays an important role in providing essential nutrients and resisting pathogenic microorganisms. For example, nitrogen synthesis (Potrikus and Breznak, 1977), degradation of plant allelochemicals (Lauzon et al., 2000), and participation in pheromone synthesis (Dillon et al., 2002). In this study, the relative abundance of *Pantoea* in male aphids was significantly higher than that in other reproductive morphs (Figures 2C, 3A), but whether *Pantoea* is related to aphid reproduction is unclear. *Enterobacter* is the dominant genus of the intestinal microbiota in most insects, playing an important role in the digestion and nutrition of insects, and it is even a protein source in *Ceratitis capitata* larval diet (Nikolouli et al., 2021). High relative abundance of *Enterobacter* in gynoparae and males (Figures 2C, 3A) might be related to the long-distance migration aimed at interpopulation gene exchanges (Huangfu et al., 2022).

UPGMA clustering analysis showed that all the samples were clustered into two categories (Supplementary Figure S2). Cluster I consisted of PF, SP, GP, and SF, while Cluster II contained only males. Notably, both PCA and UPGMA clustering results revealed that microbial community structure was more similar between sexuparae and sexual females, and between PFs and gynoparae (Figure 2A; Supplementary Figure S2). At the genus level, microbial community differed significantly in richness and diversity, but exhibited generation-skipping similarity across the reproductive morphs of cotton aphid. Furthermore, a total of 537 bacterium species were identified from all the samples, of which 75 species were shared by all the five reproductive morphs. Males displayed the largest number of unique bacterial species (109 species), followed by sexual females, PFs, sexuparae, and gynoparae, with the number of 72, 25, 24, and 14, respectively (Figure 2D). Our clustering analysis showed that four females morphs (SP, SF, PF, and GP) clustered together, and they were separated from males (Supplementary Figure S2). We speculated that intergenerational similarity in four generational female morphs (PF, SP, GP, and SF) might be due to vertical transmission of bacteria and reproductive phenotype switch in cotton aphid. Our speculation was supported by some previous reports that symbiotic gut bacteria in many species including flies affect their hosts’ development and homeostasis across generations (Fridmann-Sirkis et al., 2014), and that the exposure of fly larvae to G418 antibiotic can induce trans-generationally heritable phenotypes (Fridmann-Sirkis et al., 2014). Taken together, reproductive morph switch induced by environmental changes (low temperature and short photoperiod) might be associated with bacterial community variation and sexual dimorphism of aphids.

### 3.3. Important symbiotic bacteria and reproductive morph transformation in *Aphis gossypii*

*Buchnera* was the dominant genus, exhibiting the highest abundance among all the five reproductive morphs of *A*. *gossypii*, followed by *Rhodococcus*, *Pseudonocardia*, *Pantoea*, *Enterobacter*, *Pseudomonas*, *Microbacteria*, and *Arsenophonus* (Figure 3A; Supplementary Table S3). The relative abundance of these bacterial genera differed among different reproductive morphs. We analyzed the symbiotic bacterium changes in *A*. *gossypii* according to sexual females and males production process (PF-SP-GP-SF and PF-SP-M), respectively (Supplementary Figures S3, S4).

In the PF-SP-GP-SF reproductive switch process (PFs to sexual females), dynamic changes of symbiotic bacterial abundance were presented at different taxonomic levels (Supplementary Figure S3). Generally, at the genus level, the relative abundance of *Rhodococcus* differed significantly among four morphs, but differed slightly between PFs (0.43%) and sexuparae (0.44%). Gynoparae exhibited the highest relative abundance (1.18%) in PF-SP-GP-SF reproductive switch process, and sexual females ranked the second (0.54%). *Enterobacter*, *Dolosigranulum*, and *Cyanobacteria* showed similar trend to *Rhodococcus*. The relative abundance of *Arsenophonus* showed a consistently increasing trend in PF-SP-GP reproductive switch process, but it was significantly lower in sexual females (SF) than in other three morphs. The relative abundance of *Comamonadaceae* was the highest in PF. The relative abundance of primary symbiotic bacterium *Buchnera* was essentially similar among four morphs (Supplementary Figure S3; Supplementary Table S4).

In the PF-SP-M reproductive switch process (PFs to males), the symbiotic bacteria differed greatly (Supplementary Figure S4; Supplementary Table S5). At the phylum level, the relative abundance of the Proteobacteria was not significantly different from PF to SP, but it was significantly decreased in males (grandson). The relative abundance of Actinobacteria was decreased from PF to SP, whereas it was significantly increased in males. The relative abundance of Bacteriodetes showed an increasing trend along PF-SP-M switch process from PFs to males. At the genus level, the relative abundance of the aphid primary symbiont *Buchnera* was much lower in males than in PF with SP. The abundance of *Arsenophonus* in PF (grandmother) was nearly zero, whereas in SP (mother) and males (grandson) were 0.1 and 0.03%, respectively. Besides, the abundances of *Pantoea* and *Pseudonocardia* were significantly higher in males (2.22, 2.04%) than in their grandmother PF (0.01, 0.33%) and mother SP (0.04, 0.10%). Furthermore, *Acinetobacter*, *Sphingomonadales*, *Limnobacter* had higher relative abundance in PF than in its next generation SP (nearly close to zero), and the relative abundance of these three bacterial genera was the highest in males (Supplementary Figure S4; Supplementary Table S5).

### 3.4. Microbiome diversity of sexual dimorphism in *Aphis gossypii*

Symbiotic bacterial diversity of sexuparae, gynoparae, and males was examined at different taxonomic levels (Supplementary Figure S5; Supplementary Table S6). At the phylum level, compared to their mother sexuparae, gynoparae and males exhibited significantly lower the relative abundance of Proteobacteria, but higher relative abundance of Actinobacteria and Firmicutes. In addition, the relative abundance of most bacterial phyla except Proteobacteria was higher in gynoparae and males than their mother sexuparae. At the genus level, the relative abundance of the primary symbiotic bacterium *Buchnera* was lower in gynoparae and males than in their mother sexuparae, whereas the relative abundance of *Dolosigranulum* in gynoparae was higher than that in mother sexuparae and brother males (Supplementary Figure S5; Supplementary Table S6).

Sexual dimorphism of cotton aphid mainly exhibited the differences in appearance of two sexes, namely, exclusive apterous sexual females and exclusive alate males (Huangfu et al., 2022). In this study, distinct sexual dimorphic microbiome diversity was observed between males and sexual females of cotton aphid (Supplementary Figure S6; Supplementary Table S7). The richness of almost all the investigated bacterium species except *Buchnera* was significantly higher in males than in sexual females. Specifically, the relative abundance of *Pseudonocardia*, *Pseudomonas*, *Arsenophonus*, and *Acinetobacter* was significantly higher in males (2.04, 1.21, 0.03, and 0.40%) than sexual females (0.33, 0.15, 0.00, and 0.01%; Supplementary Figure S6; Supplementary Table S7). Further, the relative abundance of common aphid secondary symbiotic bacterial genera (*Pseudomonas*, *Enterobacter*, *Pseudonocardia*, *Arsenophonus*, and *Acinetobacter*) was compared among the five reproductive morphs of *A*. *gossypii* (Figure 3B). The results showed that the relative abundance of *Pseudonocardia*, *Enterobacter*, *Pseudomonas*, and *Acinetobacter* were all significantly higher in males than in the other four reproductive morphs. Notably, the relative abundance of *Arsenophonus* was the highest in gynoparae, followed by sexuparae and males, and this genus was almost absent in PFs and sexual females, which was significantly different from the observation on other bacterial genera (Figure 3B). *Arsenophonus*, the male-killing bacterium, kills a large number of male embryos produced by infected females (Taylor et al., 2011). Simon’s study showed that *Spiroplasma* infection of *Acyrthosiphon pisum* eliminates male production in host offspring by inducing male-killing phenotype (Simon et al., 2011). Consistently, in *Ommatissus lybicus*, the application of antibiotics to eradicate *Arsenophonus* from three populations (Fin, Qale’e Qazi, and Roodan) resulted in complete loss of offspring (Karimi et al., 2019). In addition, *Arsenophonus* enabled the brown planthopper *Nilaparvata lugens* to thrive on a low-nutrient rice diet through vitamin B supplementation (Xue et al., 2014). Obviously, *Arsenophonus* was likely to be vertically transmitted intergenerationally. The increasing evidence indicated that *Arsenophonus* vertically transmits (VT) from parent to offspring, and VT is heritable among generations (Drew et al., 2021). One recent study has revealed that *Arsenophonus nasoniae* can undergo seven generations of vertical transmission in *Pteromalus venustu*s (Goerzen and Erlandson, 2018). High abundance of *Arsenophonus* in gynoparae (Supplementary Table S3) might contribute to their male-killing, thus leading to the production of exclusive sexual females offspring (Liu et al., 2014; Kwon and Kim, 2017; Ji et al., 2021).

### 3.5. Function prediction of bacterial community

**Figure 4:**
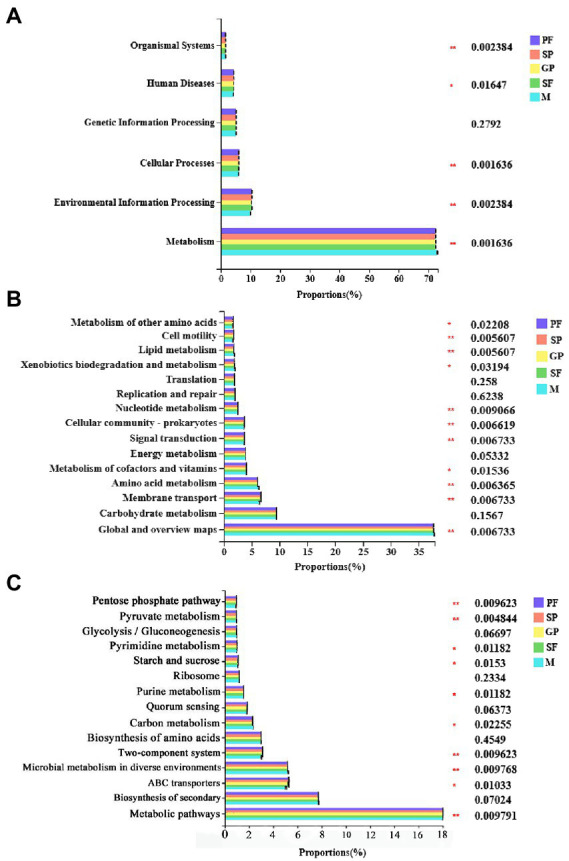
Functional prediction of symbiotic bacteria in five reproductive mode shift of cotton aphid. KEGG categories were derived from the 16S rRNA sequences of cotton aphid microbiome by PICRUSt2. **(A)** Level 1: 6 categories of biological functions of bacterial community. **(B)** Level 2: top 15 function pathways enriched with highest-richness bacteria. **(C)** Level 3: Function pathways enriched with the highest-abundance bacteria in all five morphs. Statistical significance of bacterium relative abundance proportions enriched in KEGG pathways among five reproductive morphs was determined by Tukey–Kramer test at *p* ≤ 0.05 (*) or *p* ≤ 0.01 (**) (FDR adjusted) with 95% confidence intervals. PF, parthenogenetic females; SP, sexuparae; GP, gynoparae; SF, sexual females; M, males.

The biological functions of bacteria community were predicted *via* PICRUSt2 software based on 16S rRNA sequencing data and compared against the KEGG database. The results were demonstrated at three levels. Level 1 showed 6 categories of biological functions of bacterial community including metabolism, environmental information processing, cellular processes, genetic information processing, human diseases, and organismal systems (Figure 4A; Supplementary Table S8). The relative abundance of metabolism-related bacteria was the highest (70% ~ 80%) in all the samples, followed by environmental information processing (10% ~ 15%) and cellular processes (5% ~ 10%), indicating that metabolism played an important role in the bacterial community’s response to their host’s reproductive morph switch. In *Acyrthosiphon pisum*, metabolomic experiments of several aphid genotypes, either naturally carrying or lacking *Hamiltonella*, showed that the titer of only one metabolite, 5-aminoimidazole-4-carboxamide ribonucleotide (AICAR), was nearly significantly enriched in *Hamiltonella*-positive aphids. Indicating that *Buchnera*-mediated histidine production is elevated in *Hamiltonella*-positive aphids (Blow et al., 2020). Level 2 showed the top 15 pathways enriched with highest-richness bacteria (Figure 4B; Supplementary Table S9). The bacteria with high relative abundances were found to be enriched in global and overview maps (35% ~ 40%), carbohydrate metabolism (7% ~ 10%), membrane transport (6% ~ 8%) and amino acid metabolism (6% ~ 8%) function pathways. Of these pathways, energy metabolism, replication and repair, carbohydrate metabolism and translation exhibited no significant differences among the five reproductive morphs of *A*. *gossypii*. The predicted function pathways with significant differences among these five morphs were mainly cell motility, lipid metabolism, and nucleotide metabolism (Figure 4B; Supplementary Table S9). Interestingly, males had lower membrane transport capacity but higher amino acid metabolism capacity than other reproductive morphs. At level 3, the pathways which were enriched with the highest-abundance bacteria in all five morphs mainly included metabolic pathways, biosynthesis of secondary metabolites, and ABC transporters (Figure 4C; Supplementary Table S10). Furthermore, males exhibited a lower ABC transporters capacity than other four reproductive morphs.

### 3.6. Phylogenetic analysis and quantification of *Buchnera* and *Arsenophonus*

**Figure 5:**
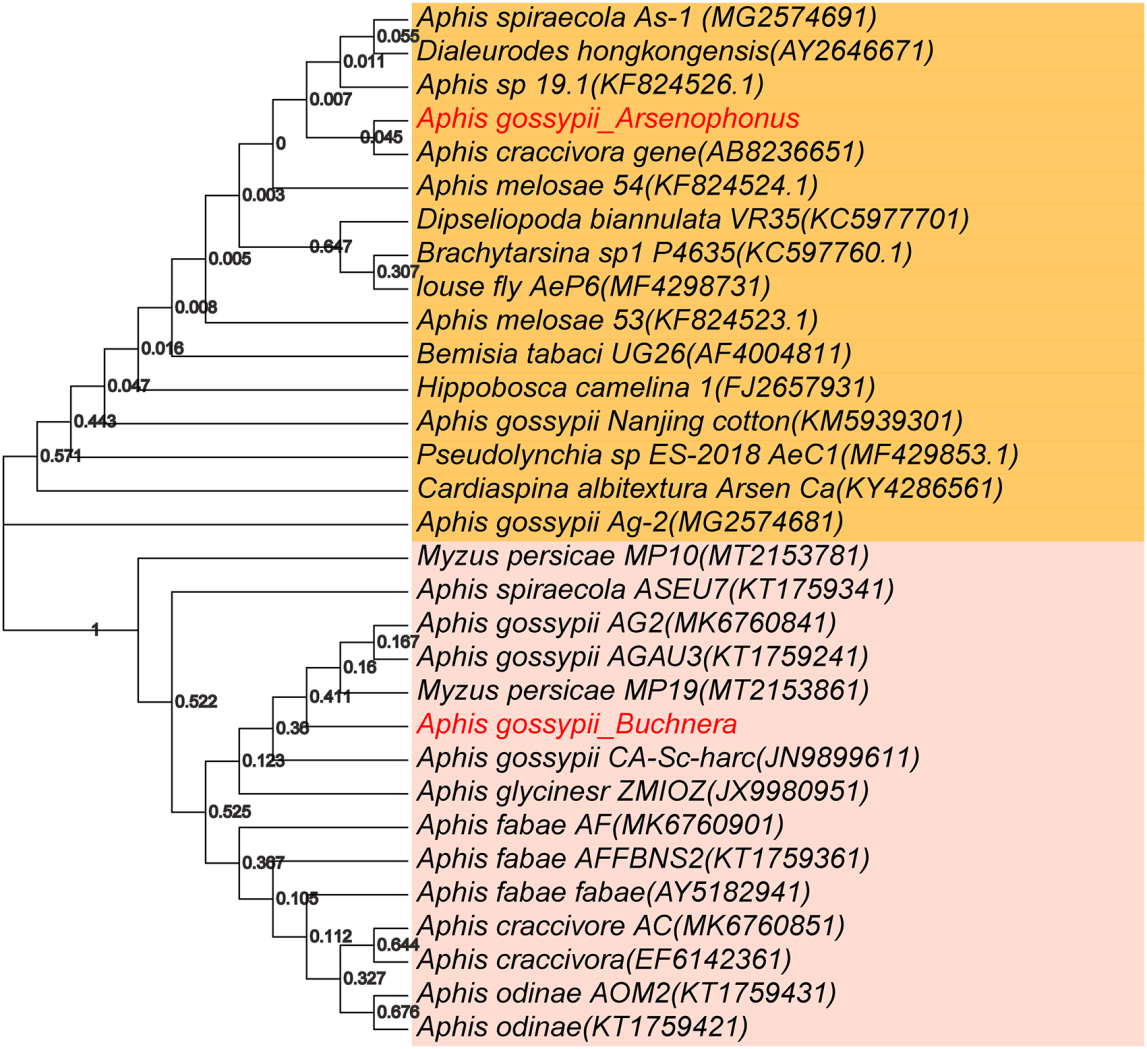
Phylogenetic tree of *Buchnera* (below) and *Arsenophonus* (above) from different host aphid species. The phylogenetic analysis was based on 31 nucleotides, and phylogenetic tree was constructed by the neighbor-joining through MEGA7.0 software with 1,000 Bootstrap replicates. GeneBank accession number of the 16S rRNA gene sequences of these two genera from different insects are listed in parentheses, respectively.

*Buchnera* is the dominant bacterium in aphids. *Arsenophonus* is a well-known as male-killing symbiotic bacterium, and it can cause the death of males offspring in early development stage, eventually resulting in female-biased sex ratio in insects. The phylogenetic tree of these two important aphid bacterial genera (*Buchnera* and *Arsenophonus*) was constructed based on the 16S rRNA sequences (Figure 5). The sequences of *Arsenophonus* in *A*. *gossypii* were highly similar to those in *A*. *craccivora*, indicating a close relationship between *A*. *gossypii* in this study and *A*. *craccivora*. Additionally, the sequences of *Arsenophonus* were also similar among *A*. *spiraecola*, *Aphis* sp. 19.1, and *Dialeurodes hongkongensis*. However, phylogenetic tree showed that our obtained sequences of *Arsenophonus* in *A*. *gossypii* were distant from those in Nanjing cotton *A*. *gossypii* and *A*. *gossypii* Ag-2, which might be due to the difference in geographical location. The sequences of *Buchnera* measured in this study exhibited a high similarity to those in *Myzus persicae* MP19, *A*. *gossypii* AGAU3 and *A*. *gossypii* AG2, but a relatively low similarity to those in *A*. *fabae*, *A*. *craccivora* and *A*. *odinae*.

**Figure 6:**
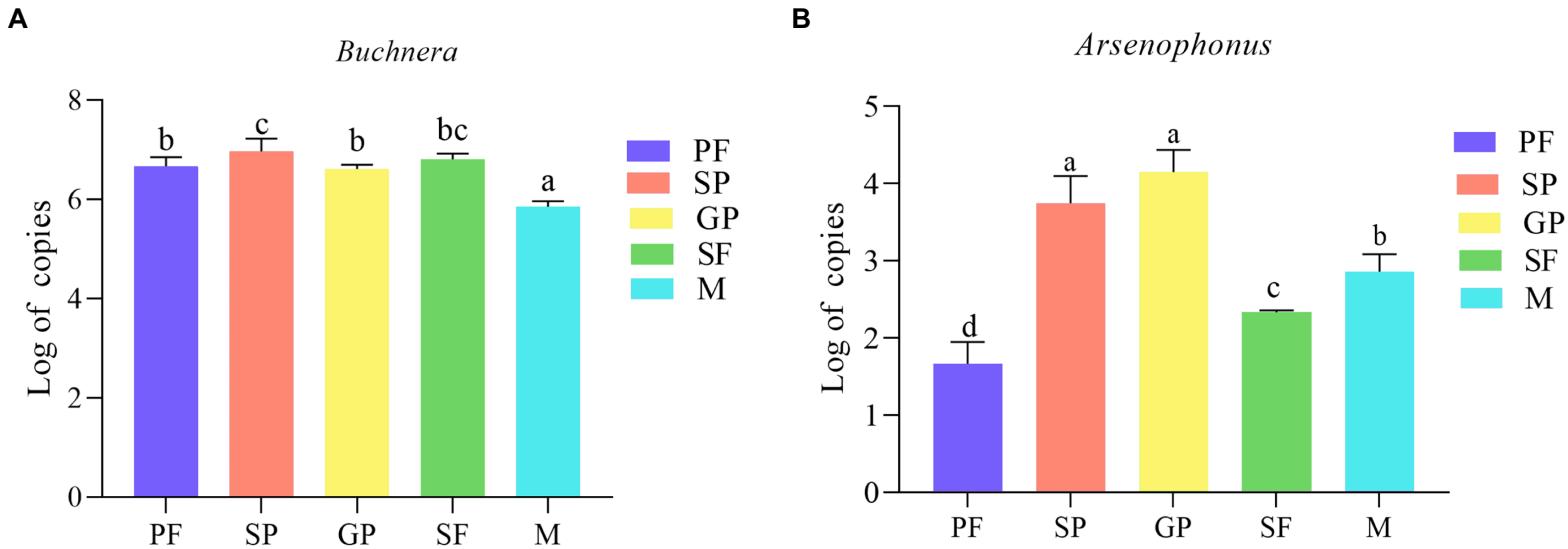
Copy number of *Buchnera* **(A)** and *Arsenophonus* **(B)** in five *A*. *gossypii* reproductive morphs. Copy number is represented by logarithmic 10 transformed value (Log 10). Significant differences were examined via one-way ANOVA. Columns (Mean ± SE) with the same letters are not significantly different (Duncan test; *p* > 0.05). PF, parthenogenetic females; SP, sexuparae; GP, gynoparae; SF, sexual females; M, males.

Quantitative PCR results (compared by one-way ANOVA) showed that the copies of *Arsenophonus* were much less than those of *Buchnera* in all the five morphs (Figure 6). During the switch from asexual reproduction (PF) to sexual reproduction (SP and GP), the copy number of *Arsenophonus* increased significantly (Figure 6B). During sexual reproduction, the copy number of *Arsenophonus* in SP and GP was significantly higher than that in SF and males (Figure 6B). Similar to our results, the female-biased offspring sex ratio produced by sexuparae (approximate 14 GP and 9 males produced by each sexupara) of *A*. *gossypii* and exclusive sexual females offspring produced by gynopare (Ji et al., 2021). The verification of *Buchnera* and *Arsenophonus* copy number is consistent with our above results (Figure 3A; Supplementary Table S3). *Buchnera* is abundant in aphids and may be used for growth and metabolism. The changes of *Arsenophonus* may be related to reproduction and may be a factor affecting reproductive polymorphism.

## 4. Conclusion

The microbial communities were dynamic, diverse, sex-biased, and different between sexes. Proteobacteria was the dominant phylum in all reproductive morphs, *Buchnera* and *Rhodococcus* were the dominant bacterium genera. The species richness and community diversity of males were significantly higher than those females of *A*. *gossypii*. The secondary symbiotic bacterium *Arsenophonus* existing in all the females exhibited the highest relative abundance in gynoparae whose offspring was exclusively males, suggesting its male-killing functions. In addition, the functions of the cotton aphid symbiotic bacteria were mainly related to metabolism. Overall, our findings provide a new perspective to reveal the interaction between bacterial community and reproductive polymorphism of aphids.

## Data availability statement

The datasets presented in this study can be found in online repositories. The names of the repository/repositories and accession number(s) can be found in the article/Supplementary material.

## Author contributions

JJ, XM, and JC conceived and designed the research. JJ, RC, and NH conducted experiments. XZ, LW, JL, and DL collected samples. JJ, RC, KZ, XG, and LN analyzed the data. JJ and RC wrote the manuscript. All authors have read and approved the manuscript.

## Funding

This research was funded by National Natural Science Foundation of China (No. 32102214) and Special Fund for Basic Public Welfare Research of Institute of Cotton Research of CAAS (No. 1610162022034). Studies were carried out in laboratories at the Institute of Cotton Research of Chinese Academy of Agricultural Sciences.

## Conflict of interest

The authors declare that the research was conducted in the absence of any commercial or financial relationships that could be construed as a potential conflict of interest.

## Publisher’s note

All claims expressed in this article are solely those of the authors and do not necessarily represent those of their affiliated organizations, or those of the publisher, the editors and the reviewers. Any product that may be evaluated in this article, or claim that may be made by its manufacturer, is not guaranteed or endorsed by the publisher.

## Footnotes

^1^ http://rdp.cme.msu.edu/classifier/classifier.jsp
