# Supplementary material for: Dynamic changes in species richness and community diversity of symbiotic bacteria in five reproductive morphs of cotton aphid Aphis gossypii Glover (Hemiptera: Aphididae)
Source: Front Microbiol. 2023 Jan 12;13:1086728. doi: 10.3389/fmicb.2022.1086728 (PMC9877530; doi:10.3389/fmicb.2022.1086728)
Supplement: Supplementary file 1 [file Data_Sheet_1.docx]

**Dynamic changes in species richness and community diversity of symbiotic bacteria in five reproductive morphs of Cotton Aphid *Aphis gossypii* Glover (Hemiptera: Aphididae)**

Ruifang Chen^1,2^, Junyu Luo^1,2,3^, Xiangzhen Zhu^2^, Li Wang^2^, Kaixin Zhang^2,3^, Dongyang Li^2^, Xueke Gao^1,2,3^, Lin Niu^2^, Ningbo Huangfu^2^, Xiaoyan Ma^1,2,3^, Jichao Ji^1,2,3^*, Jinjie Cui^1,2,3^*

(*^1^ Zhengzhou Research Base, State Key Laboratory of Cotton Biology, School of Agricultural Sciences, Zhengzhou University, Zhengzhou 450001, China*

*^2^* *State Key Laboratory of Cotton Biology, Institute of Cotton Research, Chinese Academy of Agricultural Sciences, Anyang 455000, China*

*^3^ Western Agricultural Research Center, Chinese Academy of Agricultural Sciences, Changji 831100, China)*

*Correspondence to:*

*Jichao Ji,hnnydxjc@163.com;*

*Jinjie Cui, aycuijinjie@163.com*

**Table of contents**

**Fig.**

Fig. S1 α diversity index box diagram. (A) ACE index box diagram. (B) Chao1 index box diagram. (C) Coverage index box diagram. (D) Simpson index box diagram. Different letters represented significant differences (*P* < 0.05) based on one-way ANOVA test.

Fig. S2 The microbial community structure was analyzed by unweighted pair-group arithmetic average (UPGMA) method based on 16S rRNA gene amplification sequence data.

Fig. S3 Comparison of the relative abundance of symbiotic bacteria at different classification levels from the perspective of female reproductive mode switch in *A. gossypii* (PF-SP-GP-SF). (A) Phylum level. (B) Class level. (C) Order level. (D) Family level. (E) Genus level. Others, symbiotic bacteria with the relative abundance <0.1% PF, parthenogenetic females; SP, sexupara; GP, gynopara; SF, sexual female; M, male.

Fig.S4 Comparison of the relative abundance of symbiotic bacteria at different classification levels from the perspective of male production process in *A. gossypii* (PF-SP-M). (A) Phylum level. (B) Class level. (C) Order level. (D) Family level. (E) Genus level. Others, symbiotic bacteria with the relative abundance <0.1%. PF, parthenogenetic females; SP, sexupara; GP, gynopara; SF, sexual female; M, male.

Fig.S5 Comparison of the relative abundance of symbiotic bacteria at different classification levels from the perspective of mother and sister/brother in *A. gossypii* (SP-GP-M). (A) Phylum level. (B) Class level. (C) Order level. (D) Family level. (E) Genus level. Others, symbiotic bacteria with the relative abundance <0.1%.PF, parthenogenetic females; SP, sexupara; GP, gynopara; SF, sexual female; M, male.

Fig. S6 Comparison of the symbiotic bacteria at different classification levels from the perspective of sexual dimorphism (M-SF). (A) Phylum level. (B) Class level. (C) Order level. (D) Family level. (E) Genus level. Others, symbiotic bacteria with the relative abundance <0.1%.PF, parthenogenetic females; SP, sexupara; GP, gynopara; SF, sexual females; M, male.

**Table.**

Table S1 Summary of 16S rRNA sequencing results

Table S2 Relative abundance of bacteria communities at the phylum level in different group

Table S3 Relative abundance of bacteria communities at the genus level in different group (Top 15)

Table S4 Relative abundance of bacteria communities at the genus level in PF-SP-GP-SF

Table S5 Relative abundance of bacteria communities at the genus level in PF-SP-M

Table S6 Relative abundance of bacteria communities at the genus level in SP-GP-M

Table S7 Relative abundance of bacteria communities at the genus level in males and sexual females

Table S8 Functional prediction of bacterial community at Level 1

Table S9 Functional prediction of bacterial community at Level 2

Table S10 Functional prediction of bacterial community at Level 3


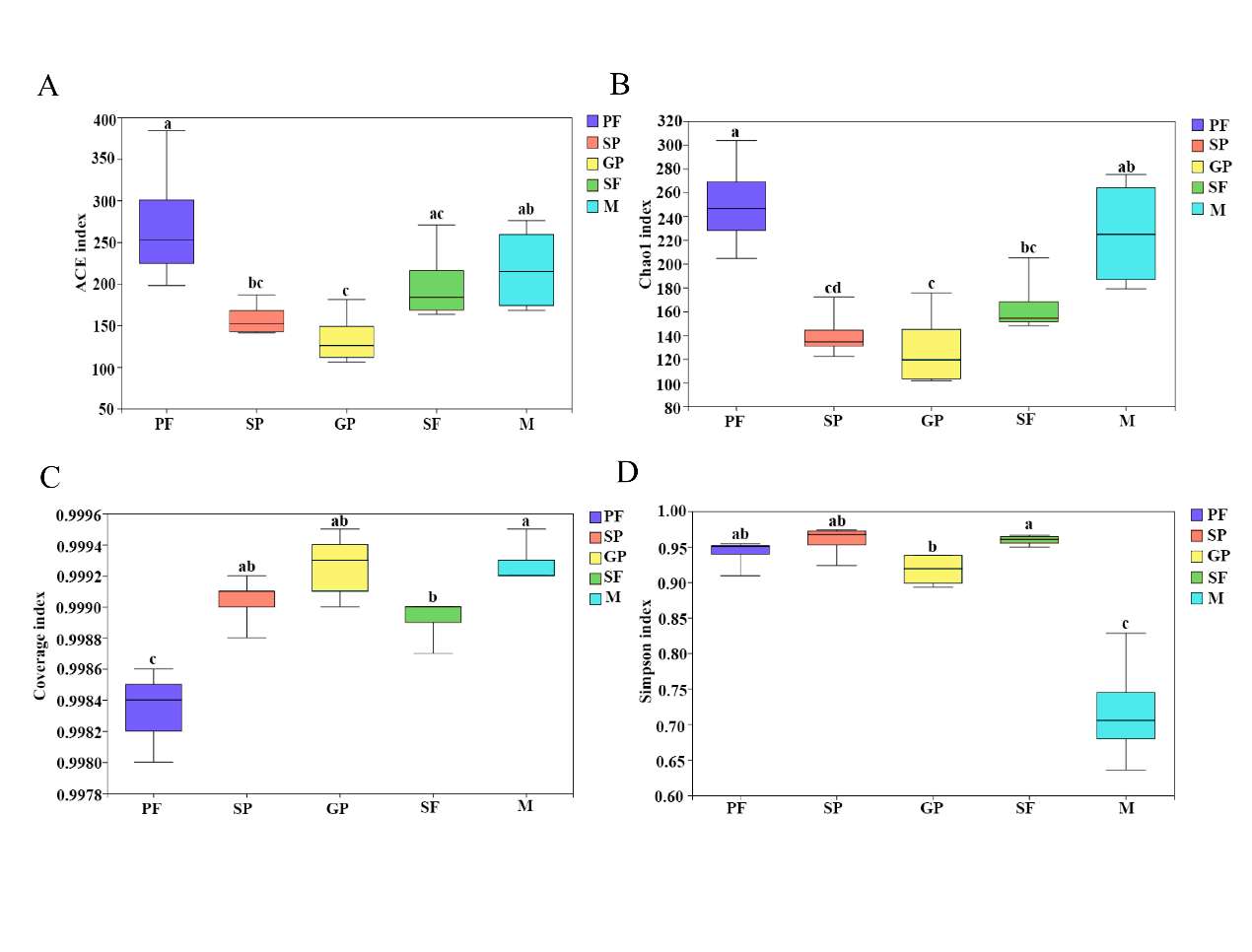


Fig. S1 α diversity index box diagram. (A) ACE index box diagram. (B) Chao1 index box diagram. (C) Coverage index box diagram. (D) Simpson index box diagram. Different letters represented significant differences (*P* < 0.05) based on one-way ANOVA test.


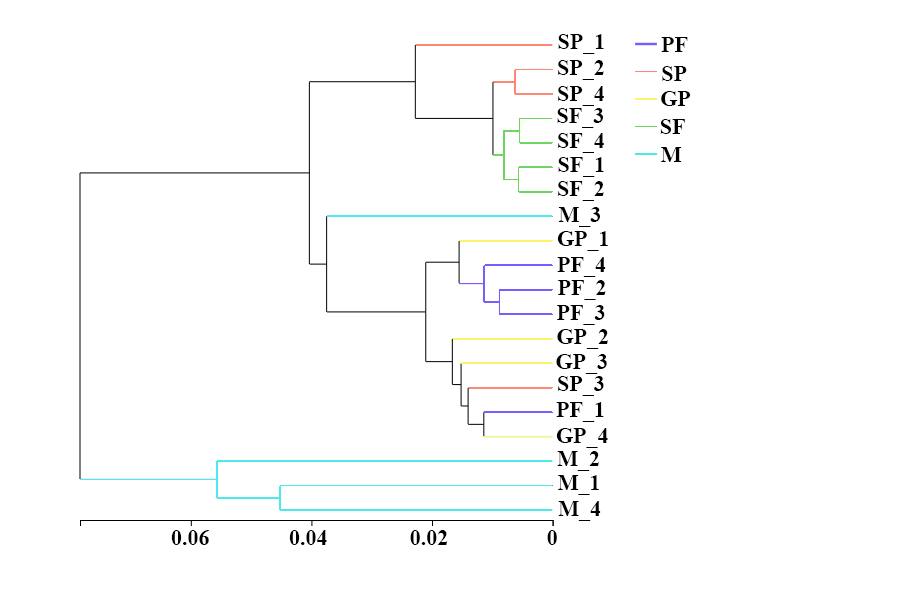


Fig. S2 The microbial community structure was analyzed by unweighted pair-group arithmetic average (UPGMA) method based on 16S rRNA gene amplification sequence data.


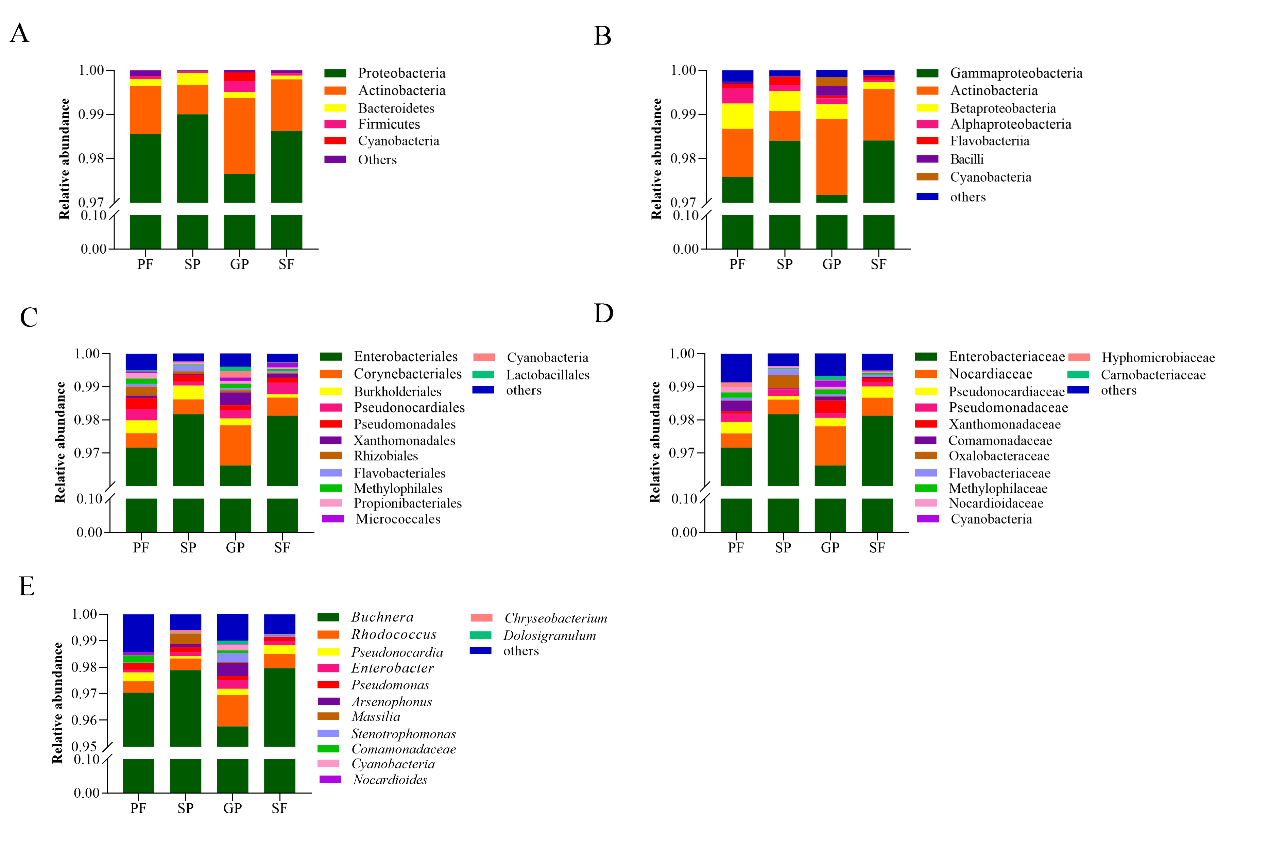


Fig. S3 Comparison of the relative abundance of symbiotic bacteria at different classification levels from the perspective of female reproductive mode switch in *A. gossypii* (PF-SP-GP-SF). (A) Phylum level. (B) Class level. (C) Order level. (D) Family level. (E) Genus level. Others, symbiotic bacteria with the relative abundance <0.1% PF, parthenogenetic females; SP, sexupara; GP, gynopara; SF, sexual female; M, male.


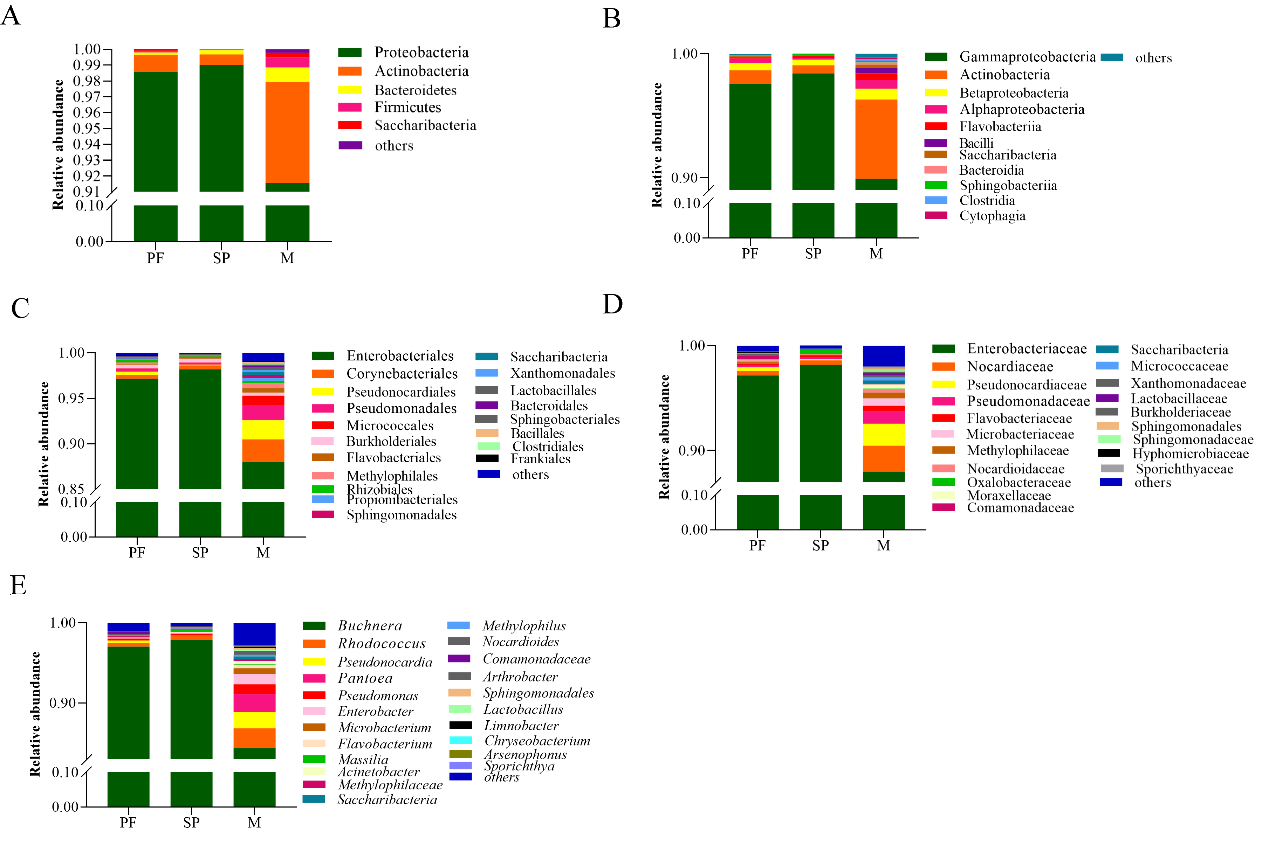


Fig.S4 Comparison of the relative abundance of symbiotic bacteria at different classification levels from the perspective of male production process in *A. gossypii* (PF-SP-M). (A) Phylum level. (B) Class level. (C) Order level. (D) Family level. (E) Genus level. Others, symbiotic bacteria with the relative abundance <0.1%.

PF, parthenogenetic females; SP, sexupara; GP, gynopara; SF, sexual female; M, male.


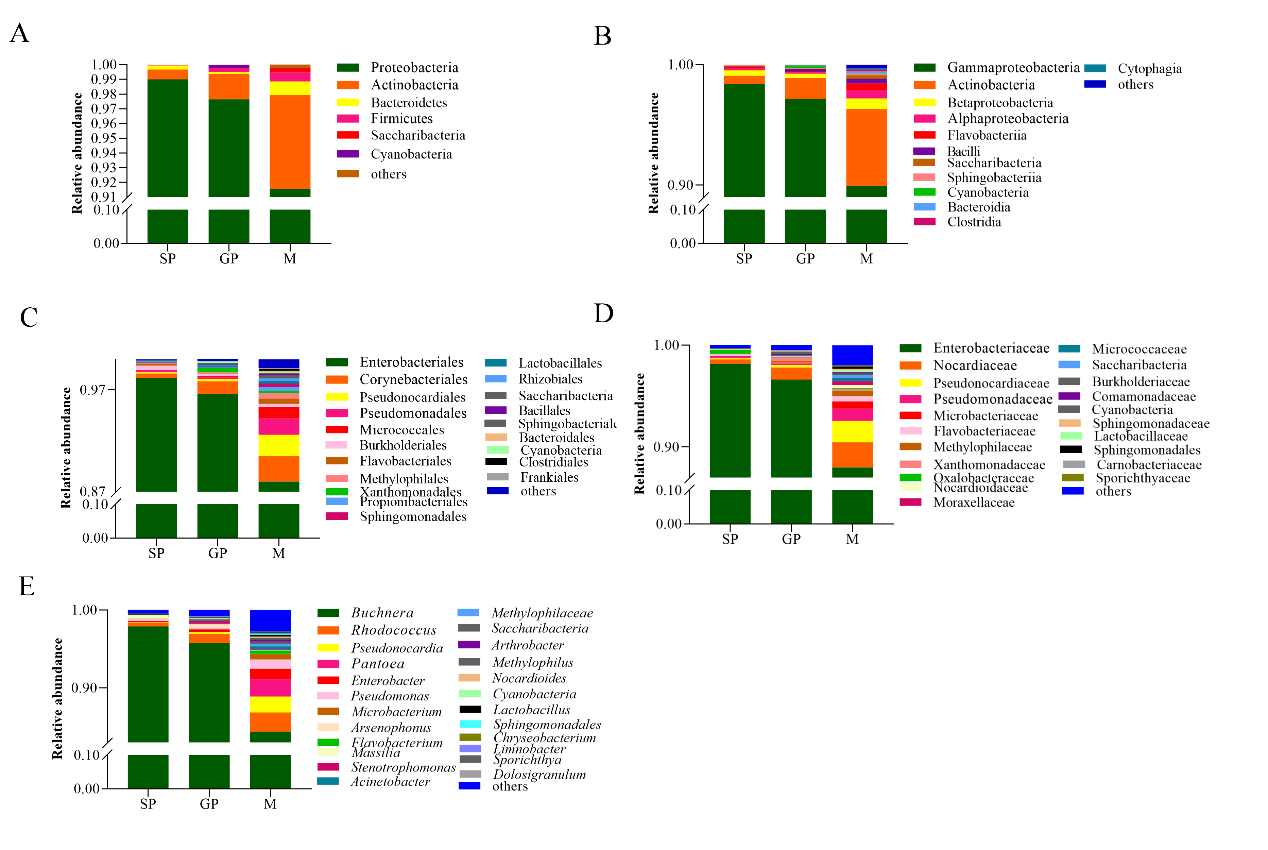


Fig.S5 Comparison of the relative abundance of symbiotic bacteria at different classification levels from the perspective of mother and sister/brother in *A. gossypii* (SP-GP-M). (A) Phylum level. (B) Class level. (C) Order level. (D) Family level. (E) Genus level. Others, symbiotic bacteria with the relative abundance <0.1%.PF, parthenogenetic females; SP, sexupara; GP, gynopara; SF, sexual female; M, male.


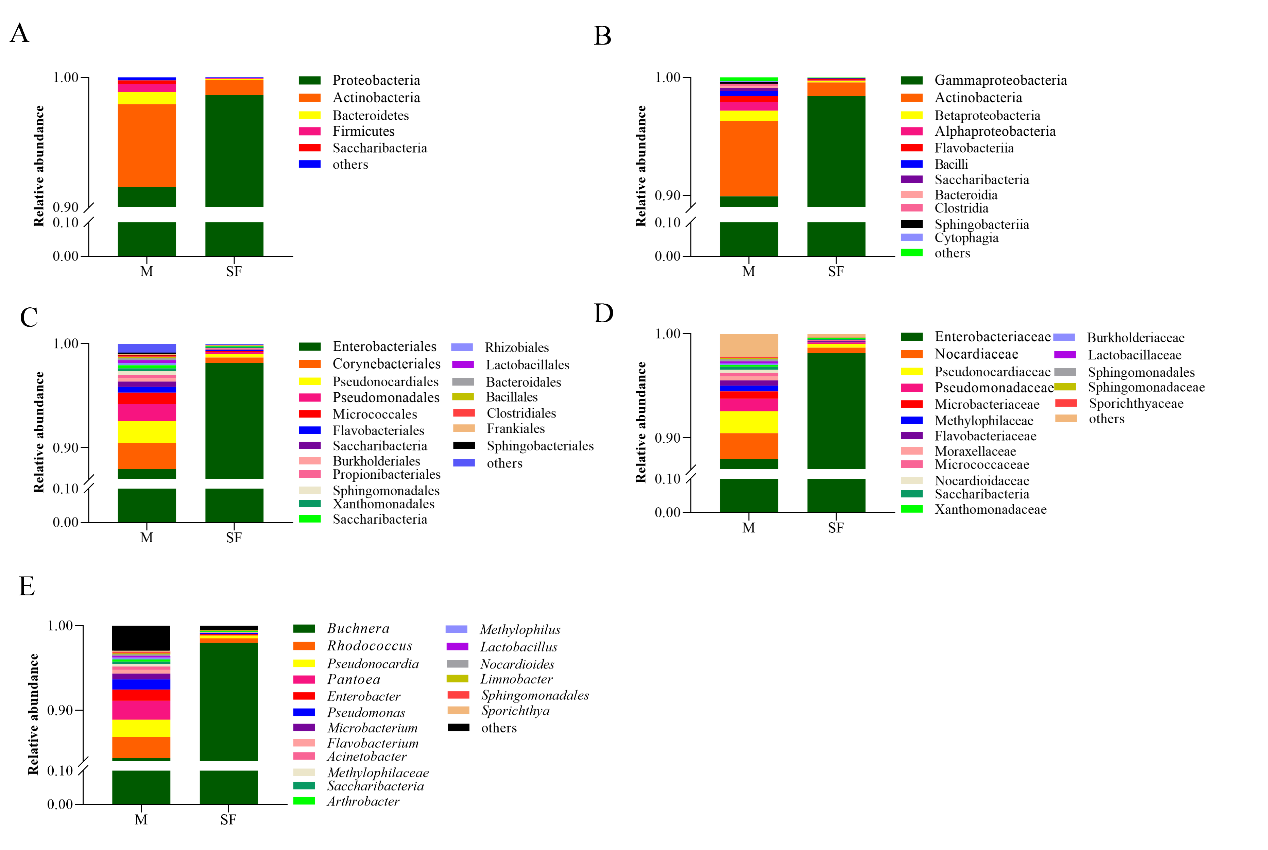


Fig. S6 Comparison of the symbiotic bacteria at different classification levels from the perspective of sexual dimorphism (M-SF). (A) Phylum level. (B) Class level. (C) Order level. (D) Family level. (E) Genus level. Others, symbiotic bacteria with the relative abundance <0.1%.PF, parthenogenetic females; SP, sexupara; GP, gynopara; SF, sexual females; M, male.

Table S1 Summary of 16S rRNA sequencing results

| Samples | Sequences  number | Mean length | OTUs | Shannon | Chao1 | ACE | Simpson | Coverage |
| --- | --- | --- | --- | --- | --- | --- | --- | --- |
| PF_1 | 40930 | 428.72 | 122 | 0.1893 | 204.65 | 197.69 | 0.9542 | 0.9986 |
| PF_2 | 40126 | 428.68 | 174 | 0.2200 | 303.6 | 383.70 | 0.9499 | 0.9980 |
| PF_3 | 39517 | 428.68 | 164 | 0.2182 | 235.72 | 233.42 | 0.9504 | 0.9983 |
| PF_4 | 40330 | 428.41 | 225 | 0.3892 | 257.18 | 272.64 | 0.9091 | 0.9985 |
| SP_1 | 44309 | 428.73 | 108 | 0.2785 | 133.87 | 143.21 | 0.9234 | 0.9992 |
| SP_2 | 44267 | 428.85 | 81 | 0.1150 | 122.05 | 140.85 | 0.9717 | 0.9991 |
| SP_3 | 41545 | 428.62 | 88 | 0.1125 | 172.00 | 186.75 | 0.9735 | 0.9988 |
| SP_4 | 45359 | 428.62 | 107 | 0.1522 | 134.77 | 161.18 | 0.9622 | 0.9991 |
| GP_1 | 39255 | 428.76 | 74 | 0.2081 | 101.56 | 113.60 | 0.9372 | 0.9992 |
| GP_2 | 42410 | 428.41 | 142 | 0.3857 | 175.44 | 180.87 | 0.8931 | 0.9990 |
| GP_3 | 42540 | 428.45 | 123 | 0.3560 | 134.54 | 137.93 | 0.9007 | 0.9994 |
| GP_4 | 40733 | 428.58 | 91 | 0.2244 | 103.83 | 105.66 | 0.9377 | 0.9995 |
| SF_1 | 45722 | 428.61 | 146 | 0.2047 | 205.00 | 270.75 | 0.9496 | 0.9987 |
| SF_2 | 45463 | 428.79 | 104 | 0.1435 | 155.75 | 197.33 | 0.9660 | 0.9990 |
| SF_3 | 46071 | 428.73 | 113 | 0.1727 | 152.60 | 163.09 | 0.9571 | 0.9990 |
| SF_4 | 46314 | 428.73 | 117 | 0.1519 | 147.94 | 170.11 | 0.9637 | 0.9990 |
| M_1 | 41704 | 426.96 | 261 | 1.1025 | 275.10 | 275.85 | 0.6940 | 0.9992 |
| M_2 | 44851 | 427.53 | 164 | 1.0697 | 189.30 | 175.92 | 0.6356 | 0.9995 |
| M_3 | 40388 | 427.96 | 148 | 0.5793 | 179.00 | 167.94 | 0.8282 | 0.9992 |
| M_4 | 41466 | 426.47 | 235 | 1.0152 | 260.14 | 253.44 | 0.7166 | 0.9992 |

Notes: PF, parthenogenetic female; SP, sexupara; GP, gynopara; SF, sexual female; M, male.

Table S2 Relative abundance of bacteria communities at the phylum level in different group

|  | PF | SP | GP | M | SF |
| --- | --- | --- | --- | --- | --- |
| Proteobacteria | 0.98560 | 0.99003 | 0.97647 | 0.91562 | 0.98628 |
| Actinobacteria | 0.01091 | 0.00671 | 0.01722 | 0.06376 | 0.01165 |
| Bacteroidetes | 0.00145 | 0.00266 | 0.00140 | 0.00924 | 0.00085 |
| Firmicutes | 0.00071 | 0.00023 | 0.00247 | 0.00615 | 0.00053 |
| Saccharibacteria | 0.00069 | 0.00012 | 0.00022 | 0.00290 | 0.00037 |
| Cyanobacteria | 6.93E-05 | 7.49E-05 | 0.00192 | 0.00025 | 0.00011 |
| others | 0.00055 | 0.00014 | 0.00027 | 0.00205 | 0.00018 |

Notes: PF, parthenogenetic female; SP, sexupara; GP, gynopara; SF, sexual female; M, male.

Table S3 Relative abundance of bacteria communities at the genus level in different group (Top 15)

|  | GP | SF | M | SP | PF |
| --- | --- | --- | --- | --- | --- |
| *Buchnera* | 0.95714 | 0.97923 | 0.84305 | 0.97863 | 0.96961 |
| *Rhodococcus* | 0.01183 | 0.00540 | 0.02479 | 0.00437 | 0.00430 |
| *Pseudonocardia* | 0.00243 | 0.00334 | 0.02042 | 0.00096 | 0.00332 |
| *Pantoea* | 4.91E-05 | 2.31E-06 | 0.02224 | 0.00036 | 5.04E-05 |
| *Enterobacter* | 0.00341 | 0.00160 | 0.01315 | 0.00131 | 0.00105 |
| *Pseudomonas* | 0.00135 | 0.00145 | 0.01207 | 0.00205 | 0.00251 |
| *Microbacterium* | 7.36E-05 | 8.80E-05 | 0.00683 | 2.31E-05 | 0.00012 |
| *Flavobacterium* | 0.00027 | 0.00052 | 0.00440 | 0.00059 | 0.00080 |
| *Arsenophonus* | 0.00508 | 2.31E-0 | 0.00028 | 0.00115 | 2.31E-06 |
| *Stenotrophomonas* | 0.00348 | 0.00054 | 0.00096 | 2.31E-06 | 0.00012 |
| *Massilia* | 0.00022 | 4.40E-05 | 0.00057 | 0.00389 | 2.52E-05 |
| *Methylophilaceae* | 0.00063 | 0.00024 | 0.00307 | 0.00016 | 0.00070 |
| *Saccharibacteria* | 0.00022 | 0.00037 | 0.00291 | 0.000138 | 0.00068 |
| *Acinetobacter* | 2.45E-05 | 0.00013 | 0.00394 | 2.31E-06 | 7.56E-05 |
| *Comamonadaceae* | 0.00095 | 0.0002638 | 0.00014 | 4.61E-05 | 0.00274 |

Notes: PF, parthenogenetic female; SP, sexupara; GP, gynopara; SF, sexual female; M, male.

Table S4 Relative abundance of bacteria communities at the genus level in PF-SP-GP-SF

|  | PF | SP | GP | SF |
| --- | --- | --- | --- | --- |
| *Buchnera* | 0.97041 | 0.97890 | 0.95764 | 0.97964 |
| *Rhodococcus* | 0.00430 | 0.00437 | 0.01183 | 0.00541 |
| *Pseudonocardia* | 0.00333 | 0.00097 | 0.00243 | 0.00333 |
| *Enterobacter* | 0.00106 | 0.00131 | 0.00340 | 0.00160 |
| *Pseudomonas* | 0.00252 | 0.00205 | 0.00134 | 0.00145 |
| *Arsenophonus* | 0.00000 | 0.00116 | 0.00507 | 0.00000 |
| *Massilia* | 0.00001 | 0.00390 | 0.00023 | 0.00003 |
| *Stenotrophomonas* | 0.00013 | 0.00001 | 0.00348 | 0.00055 |
| *Comamonadaceae* | 0.00275 | 0.00005 | 0.00095 | 0.00026 |
| *Cyanobacteria* | 0.00006 | 0.00005 | 0.00192 | 0.00010 |
| *Nocardioides* | 0.00121 | 0.00010 | 0.00033 | 0.00020 |
| *Chryseobacterium* | 0.00002 | 0.00111 | 0.00021 | 0.00006 |
| *Dolosigranulum* | 0.00000 | 0.00000 | 0.00123 | 0.00000 |
| others | 0.01421 | 0.00602 | 0.00995 | 0.00737 |

Notes: PF, parthenogenetic female; SP, sexupara; GP, gynopara; SF, sexual female.

Table S5 Relative abundance of bacteria communities at the genus level in PF-SP-M

|  | PF | SP | M |
| --- | --- | --- | --- |
| *Buchnera* | 0.97041 | 0.97890 | 0.84375 |
| *Rhodococcus* | 0.00430 | 0.00437 | 0.02481 |
| *Pseudonocardia* | 0.00333 | 0.00097 | 0.02043 |
| *Pantoea* | 0.00004 | 0.00036 | 0.02227 |
| *Pseudomonas* | 0.00252 | 0.00205 | 0.01209 |
| *Enterobacter* | 0.00106 | 0.00131 | 0.01316 |
| *Microbacterium* | 0.00013 | 0.00002 | 0.00684 |
| *Flavobacterium* | 0.00081 | 0.00060 | 0.00441 |
| *Massilia* | 0.00001 | 0.00390 | 0.00057 |
| *Acinetobacter* | 0.00006 | 0.00000 | 0.00394 |
| *Methylophilaceae* | 0.00070 | 0.00015 | 0.00308 |
| *Saccharibacteria* | 0.00068 | 0.00013 | 0.00291 |
| *Methylophilus* | 0.00077 | 0.00014 | 0.00221 |
| *Nocardioides* | 0.00121 | 0.00010 | 0.00171 |
| *Comamonadaceae* | 0.00275 | 0.00005 | 0.00014 |
| *Arthrobacter* | 0.00009 | 0.00009 | 0.00253 |
| *Lactobacillus* | 0.00025 | 0.00008 | 0.00197 |
| *Sphingomonadales* | 0.00001 | 0.00000 | 0.00188 |
| *Limnobacter* | 0.00047 | 0.00000 | 0.00126 |
| *Chryseobacterium* | 0.00002 | 0.00111 | 0.00037 |
| *Arsenophonus* | 0.00000 | 0.00116 | 0.00028 |
| *Sporichthya* | 0.00027 | 0.00001 | 0.00109 |
| *others* | 0.01011 | 0.00451 | 0.02829 |

Notes: PF, parthenogenetic female; SP, sexupara; M, male.

Table S6 Relative abundance of bacteria communities at the genus level in SP-GP-M

|  | SP | GP | M |
| --- | --- | --- | --- |
| *Buchnera* | 0.97890 | 0.95764 | 0.84375 |
| *Rhodococcus* | 0.00437 | 0.01183 | 0.02481 |
| *Pseudonocardia* | 0.00097 | 0.00243 | 0.02043 |
| *Pantoea* | 0.00036 | 0.00004 | 0.02227 |
| *Enterobacter* | 0.00131 | 0.00340 | 0.01316 |
| *Pseudomonas* | 0.00205 | 0.00134 | 0.01209 |
| *Microbacterium* | 0.00002 | 0.00007 | 0.00684 |
| *Arsenophonus* | 0.00116 | 0.00507 | 0.00028 |
| *Flavobacterium* | 0.00060 | 0.00026 | 0.00441 |
| *Massilia* | 0.00390 | 0.00023 | 0.00057 |
| *Stenotrophomonas* | 0.00001 | 0.00348 | 0.00096 |
| *Acinetobacter* | 0.00000 | 0.00002 | 0.00394 |
| *Methylophilaceae* | 0.00015 | 0.00063 | 0.00308 |
| *Saccharibacteria* | 0.00013 | 0.00022 | 0.00291 |
| *Arthrobacter* | 0.00009 | 0.00057 | 0.00253 |
| *Methylophilus* | 0.00014 | 0.00060 | 0.00221 |
| *Cyanobacteria* | 0.00005 | 0.00192 | 0.00023 |
| *Nocardioides* | 0.00010 | 0.00033 | 0.00171 |
| *Lactobacillus* | 0.00008 | 0.00000 | 0.00197 |
| *Sphingomonadales* | 0.00000 | 0.00000 | 0.00188 |
| *Chryseobacterium* | 0.00111 | 0.00021 | 0.00037 |
| *Limnobacter* | 0.00000 | 0.00045 | 0.00126 |
| *Sporichthya* | 0.00001 | 0.00014 | 0.00109 |
| *Dolosigranulum* | 0.00000 | 0.00123 | 0.00000 |
| others | 0.00450 | 0.00789 | 0.02725 |

Note: SP, sexupara; GP, gynopara; M, male.

Table S7 Relative abundance of bacteria communities at the genus level in males and sexual females

|  | Males | Sexual females |
| --- | --- | --- |
| *Buchnera* | 0.84375 | 0.97964 |
| *Rhodococcus* | 0.02481 | 0.00541 |
| *Pseudonocardia* | 0.02043 | 0.00333 |
| *Pantoea* | 0.02227 | 0.00000 |
| *Enterobacter* | 0.01316 | 0.00160 |
| *Pseudomonas* | 0.01209 | 0.00145 |
| *Microbacterium* | 0.00684 | 0.00009 |
| *Flavobacterium* | 0.00441 | 0.00052 |
| *Acinetobacter* | 0.00394 | 0.00013 |
| *Methylophilaceae* | 0.00308 | 0.00023 |
| *Saccharibacteria* | 0.00291 | 0.00037 |
| *Arthrobacter* | 0.00253 | 0.00066 |
| *Methylophilus* | 0.00221 | 0.00018 |
| *Lactobacillus* | 0.00197 | 0.00006 |
| *Nocardioides* | 0.00171 | 0.00020 |
| *Limnobacter* | 0.00126 | 0.00057 |
| *Sphingomonadales* | 0.00188 | 0.00000 |
| *Sporichthya* | 0.00109 | 0.00021 |
| others | 0.02966 | 0.00535 |

Table S8 Functional prediction of bacterial community at Level 1

| Name | P-value | PF | SP | GP | SF | M |
| --- | --- | --- | --- | --- | --- | --- |
| Metabolism | 0.00163 | 72.510±0.034 | 72.47±0.032 | 72.59±0.060 | 72.5±0.033 | 73.11±0.129 |
| Environmental Information Processing | 0.00238 | 10.400±0.031 | 10.42±0.038 | 10.34±0.034 | 10.41±0.017 | 10.01±0.102 |
| Cellular Processes | 0.0016 | 6.101±0.008 | 6.113±0.008 | 6.069±0.016 | 6.1±0.010 | 5.925±0.040 |
| Genetic Information Processing | 0.2792 | 5.097±0.004 | 5.094±0.003 | 5.104±0.008 | 5.094±0.003 | 5.102±0.046 |
| Human Diseases | 0.0164 | 4.325±0.004 | 4.327±0.006 | 4.308±0.023 | 4.318±0.008 | 4.242±0.030 |
| Organismal Systems | 0.0023 | 1.572±0.004 | 1.571±0.007 | 1.582±0.008 | 1.571±0.002 | 1.615±0.010 |

Note: The data in the table are mean±SD. PF, parthenogenetic female; SP, sexupara; GP, gynopara; SF, sexual female; M, male.

Table S9 Functional prediction of bacterial community at Level 2

| Name | P-value | PF | SP | GP | SF | M |
| --- | --- | --- | --- | --- | --- | --- |
| Global and overview maps | 0.0067 | 37.66±0.009 | 37.64±0.008 | 37.6±0.019 | 37.6±0.010 | 37.84±0.052 |
| Carbohydrate metabolism | 0.1567 | 9.461±0.005 | 9.467±0.007 | 9.46±0.014 | 9.47±0.002 | 9.453±0.052 |
| Membrane transport | 0.0067 | 6.689±0.023 | 6.705±0.035 | 6.64±0.029 | 6.70±0.011 | 6.397±0.079 |
| Amino acid metabolism | 0.0064 | 6.066±0.017 | 6.047±0.016 | 6.08±0.019 | 6.05±0.009 | 6.291±0.054 |
| Metabolism of cofactors and vitamins | 0.0154 | 4.027±0.001 | 4.028±0.002 | 4.03±0.005 | 4.02±0.001 | 4.003±0.007 |
| Energy metabolism | 0.0533 | 3.847±0.003 | 3.842±0.001 | 3.85±0.007 | 3.84±0.001 | 3.866±0.011 |
| Signal transduction | 0.0067 | 3.706±0.008 | 3.715±0.004 | 3.69±0.011 | 3.70±0.005 | 3.608±0.027 |
| Cellular community - prokaryotes | 0.0066 | 3.703±0.007 | 3.71±0.007 | 3.68±0.011 | 3.70±0.005 | 3.586±0.030 |
| Nucleotide metabolism | 0.0091 | 2.525±0.002 | 2.529±0.002 | 2.52±0.002 | 2.52±0.001 | 2.483±0.013 |
| Replication and repair | 0.6238 | 2.015±0.000 | 2.015±0.001 | 2.01±0.002 | 2.01±0.001 | 2.011±0.0161 |
| Translation | 0.2580 | 1.889±0.002 | 1.887±0.001 | 1.89±0.004 | 1.88±0.001 | 1.896±0.021 |
| Xenobiotics biodegradation and metabolism | 0.0319 | 1.84±0.007 | 1.831±0.009 | 1.85±0.010 | 1.83±0.007 | 1.998±0.075 |
| Lipid metabolism | 0.0056 | 1.799±0.005 | 1.794±0.004 | 1.81±0.008 | 1.79±0.004 | 1.882±0.019 |
| Cell motility | 0.0056 | 1.735±0.004 | 1.744±0.005 | 1.72±0.008 | 1.73±0.004 | 1.639±0.020 |
| Metabolism of other amino acids | 0.0221 | 1.668±0.000 | 1.669±0.000 | 1.66±0.000 | 1.66±0.000 | 1.662±0.002 |

Note: The data in the table are mean±SD. PF, parthenogenetic female; SP, sexupara; GP, gynopara; SF, sexual female; M, mal

Table S10 Functional prediction of bacterial community at Level 3

| Name | P-value | PF | SP | GP | SF | | M |
| --- | --- | --- | --- | --- | --- | --- | --- |
| Metabolic pathways | 0.0097 | 18.01±0.002 | 18.01±0.002 | 18.01±0.001 | 18.01±0.000 | 17.98±0.008 | |
| Biosynthesis of secondary metabolites | 0.0702 | 7.721±0.001 | 7.719±0.000 | 7.726±0.005 | 7.723±0.002 | 7.747±0.033 | |
| ABC transporters | 0.0103 | 5.274±0.017 | 5.284±0.030 | 5.238±0.025 | 5.285±0.008 | 5.035±0.066 | |
| Microbial metabolism in diverse environments | 0.0097 | 5.156±0.003 | 5.151±0.003 | 5.164±0.013 | 5.156±0.004 | 5.234±0.020 | |
| Two-component system | 0.0096 | 3.12±0.008 | 3.128±0.004 | 3.105±0.010 | 3.123±0.000 | 3.015±0.031 | |
| Biosynthesis of amino acids | 0.4549 | 2.991±0.000 | 2.989±0.002 | 2.989±0.002 | 2.991±0.001 | 2.989±0.016 | |
| Carbon metabolism | 0.0225 | 2.286±0.003 | 2.281±0.001 | 2.29±0.004 | 2.284±0.001 | 2.323±0.016 | |
| Quorum sensing | 0.0637 | 1.84±0.001 | 1.836±0.003 | 1.831±0.005 | 1.838±0.001 | 1.809±0.018 | |
| Purine metabolism | 0.0118 | 1.534±0.001 | 1.536±0.000 | 1.534±0.001 | 1.536±0.001 | 1.514±0.006 | |
| Ribosome | 0.2334 | 1.174±0.002 | 1.172±0.001 | 1.176±0.003 | 1.172±0.001 | 1.181±0.015 | |
| Starch and sucrose metabolism | 0.0153 | 1.092±0.004 | 1.097±0.004 | 1.088±0.0036 | 1.097±0.001 | 1.052±0.015 | |
| Pyrimidine metabolism | 0.0118 | 0.991±0.001 | 0.992±0.001 | 0.9892±0.001 | 0.992±0.001 | 0.968±0.006 | |
| Glycolysis / Gluconeogenesis - | 0.0669 | 0.975±0.000 | 0.975±0.001 | 0.9775±0.002 | 0.976±0.000 | 0.981±0.009 | |
| Pyruvate metabolism | 0.0048 | 0.936±0.000 | 0.934±0.000 | 0.9384±0.001 | 0.935±0.000 | 0.953±0.003 | |
| Pentose phosphate pathway | 0.0096 | 0.943±0.003 | 0.946±0.004 | 0.9397±0.002 | 0.946±0.001 | 0.907±0.010 | |

Note: The data in the table are mean±SD. PF, parthenogenetic female; SP, sexupara; GP, gynopara; SF, sexual female; M, male.
